# Supplementary material for: Symmetric and asymmetric DNA N6-adenine methylation regulates different biological responses in Mucorales
Source: Nat Commun. 2024 Jul 18;15:6066. doi: 10.1038/s41467-024-50365-2 (PMC11258239; doi:10.1038/s41467-024-50365-2)

Supplementary information for

Symmetric and asymmetric DNA N6-adenine methylation regulates different biological responses in Mucorales

Carlos Lax, Stephen J. Mondo, Macario Osorio-Concepción, Anna Muszewska, María Corrochano-Luque, Gabriel Gutiérrez, Robert Riley, Anna Lipzen, Jie Guo, Hope Hundley, Mojgan Amirebrahimi, Vivian Ng, Damaris Lorenzo-Gutiérrez, Ulrike Binder, Junhuan Yang, Yuanda Song, David Cánovas, Eusebio Navarro, Michael Freitag, Toni Gabaldón, Igor V. Grigoriev, Luis M. Corrochano, Francisco Esteban Nicolás, Victoriano Garre

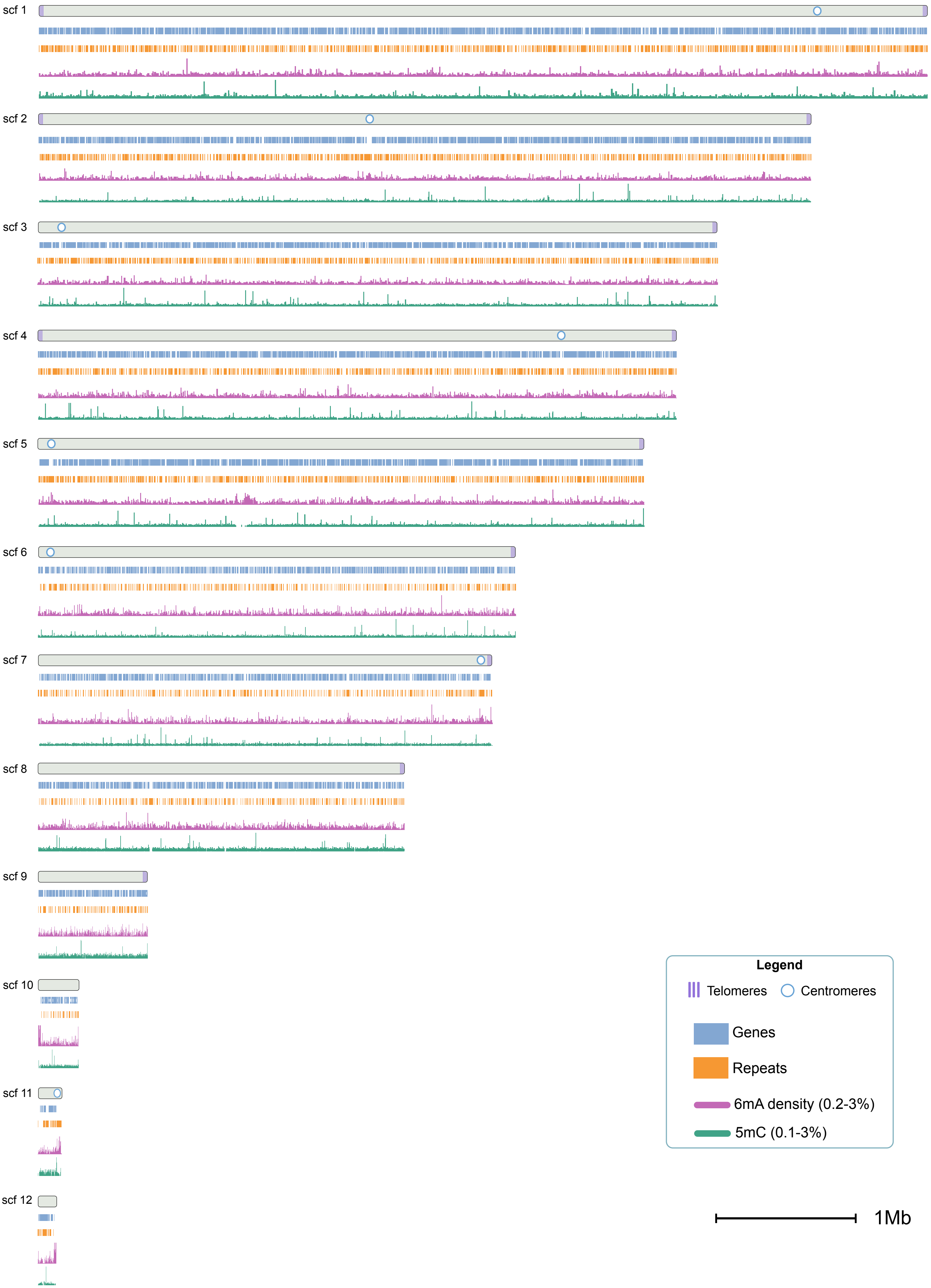

scf 1
scf 2
scf 3
scf 4
scf 5
scf 6
scf 7
scf 8
scf 9
scf 10
scf 11
scf 12
Legend
Telomeres
Centromeres
Genes
Repeats
6mA density (0.2-3%)
5mC (0.1-3%)
1Mb

Supplementary Figure 1. ***M. lusitanicus*** **DNA methylome landscape**.

Complete view of the M. *lusitanicus* genome (scaffolds 1-12) indicating gene density (blue track), repeats density (orange track) and 6mA and 5mC level (pink and green tracks, respectively). Identified telomeres and centromeres are indicated as in the legend.

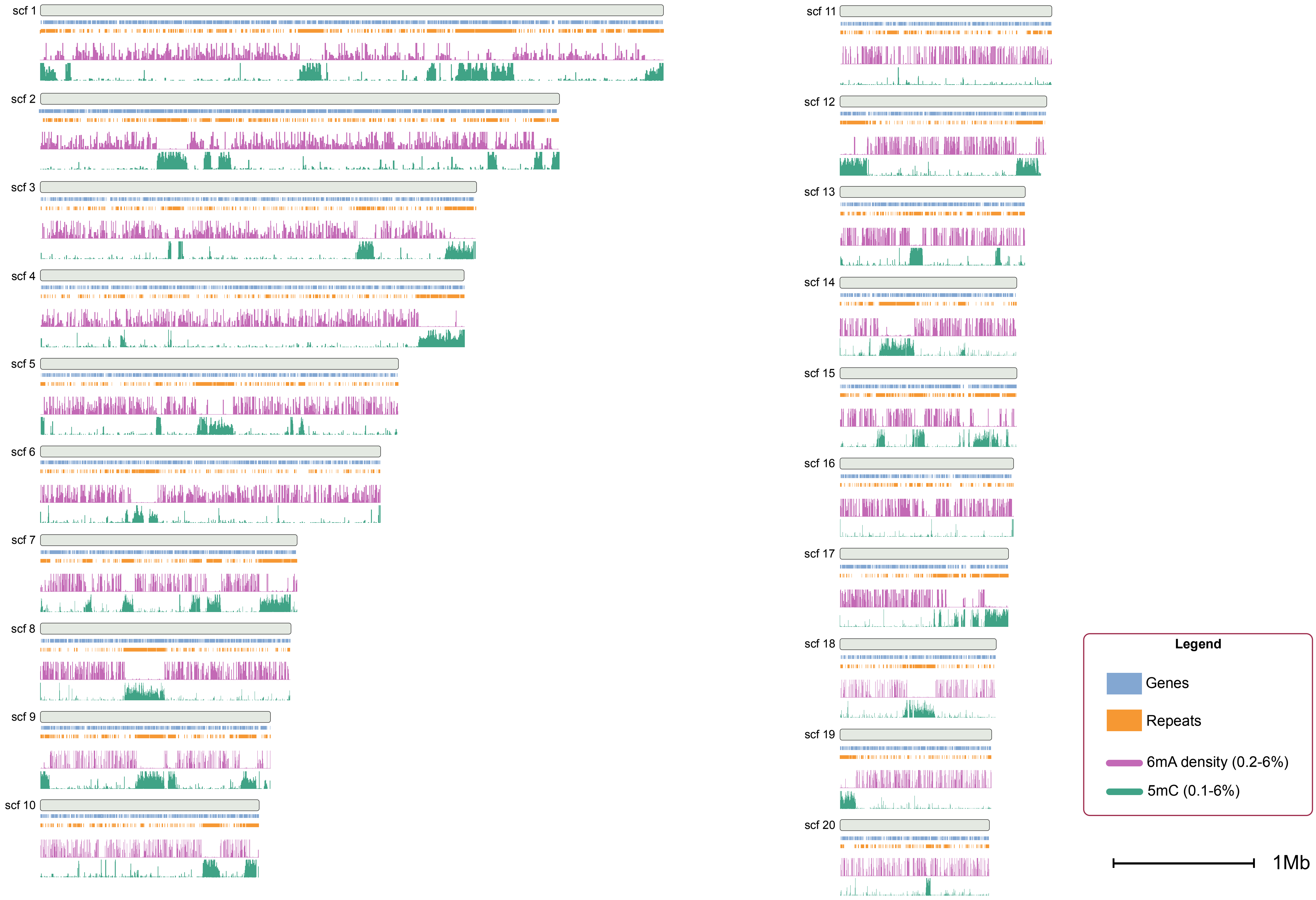

scf 1
scf 2
scf 3
scf 4
scf 5
scf 6
scf 7
scf 8
scf 9
scf 10
scf 11
scf 12
scf 13
scf 14
scf 15
scf 16
scf 17
scf 18
scf 19
scf 20
Legend
Genes
Repeats
6mA density (0.2-6%)
5mC (0.1-6%)
1Mb

Supplementary Figure 2. ***P. blakesleeanus*** **DNA methylome landscape.**

P. blakesleeanus scaffolds 1-20 indicating gene density (blue track), repeats density (orange track), 6mA (pink track) and 5mC (green track).

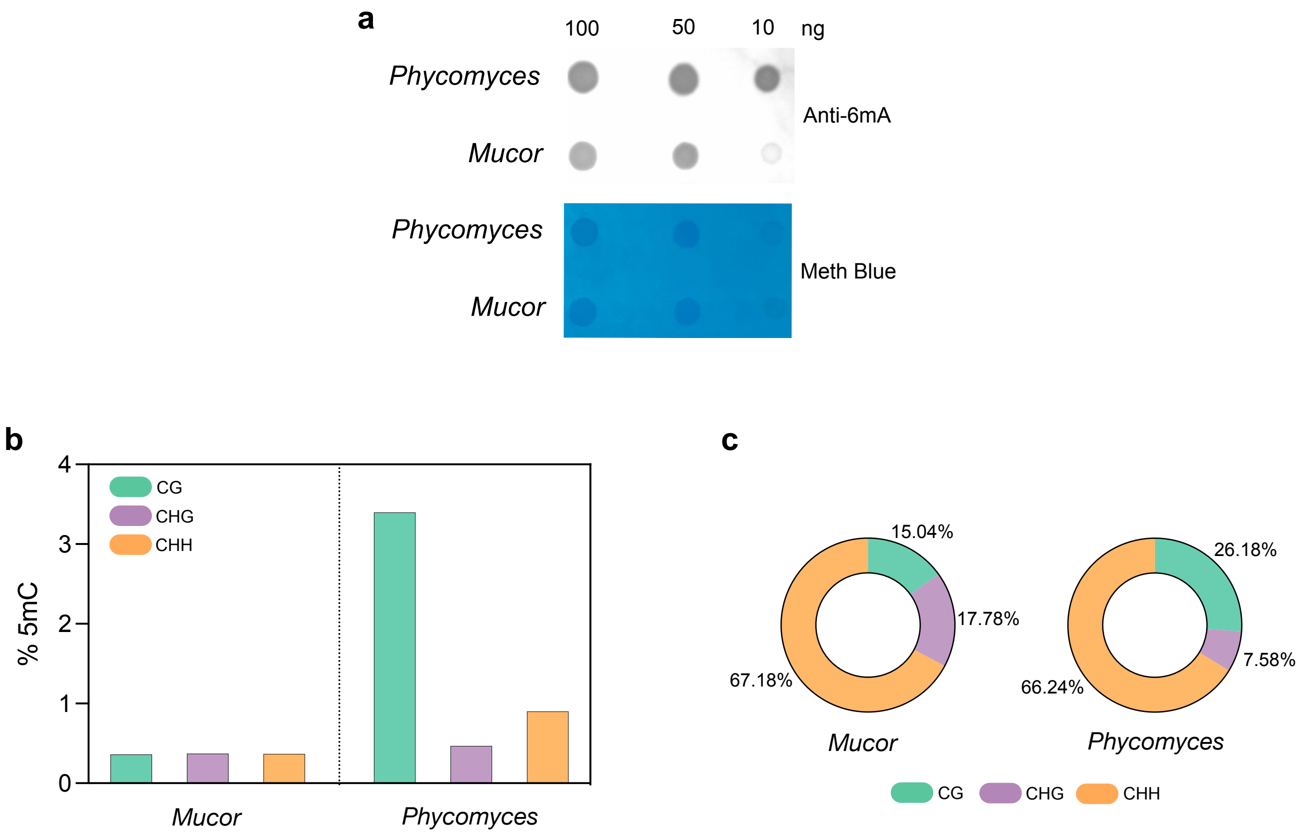

a
100
50
10
ng
Phycomyces
Mucor
Anti-6mA
Phycomyces
Mucor
Meth Blue
b
% 5mC
4
3
2
1
0
CG
CHG
CHH
Mucor
Phycomyces
c
15.04%
17.78%
67.18%
26.18%
7.58%
66.24%
Mucor
Phycomyces
CG
CHG
CHH

Supplementary Figure 3. 5mC in *M. lusitanicus* and *P. blakesleeanus*.

(A) Dot blot for 6mA detection in *P. blakesleeanus* and *M. lusitanicus* DNA. Methylene blue was used for DNA visualization on the membrane. This experiment was performed once. (B) Genomic weighted methylation levels for CG, CHG and CHH contexts (methylated reads/ methylated+unmethylated reads). (C) Proportion of 5mC sites on each context.

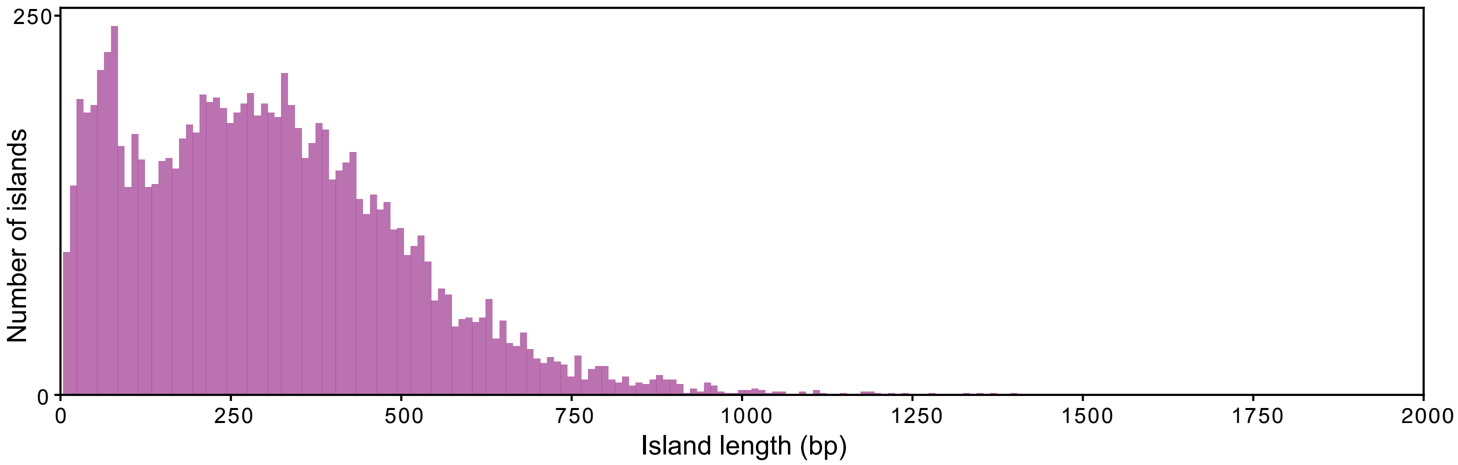

250
0
Number of islands
0
250
500
750
1000
1250
1500
1750
2000
Island length (bp)

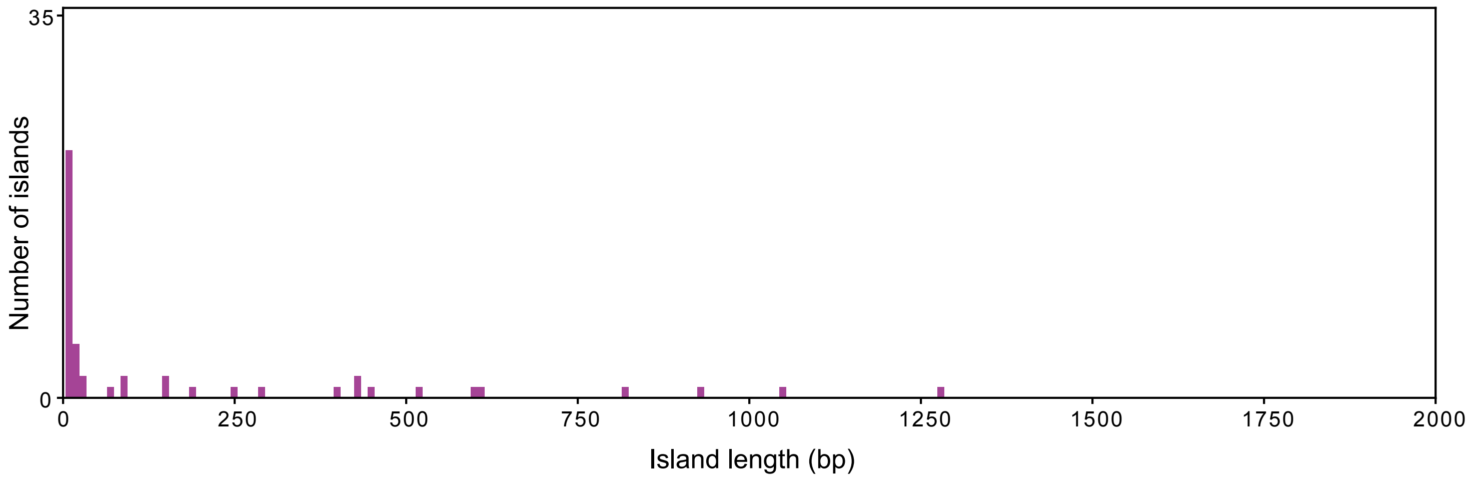

35
0
Number of islands
0
250
500
750
1000
1250
1500
1750
2000
Island length (bp)

Supplementary Figure 4. Methylated adenine clusters.

Size distribution of 6mA islands (MACs) detected in *Phycomyces* (top) and *Mucor* (bottom).

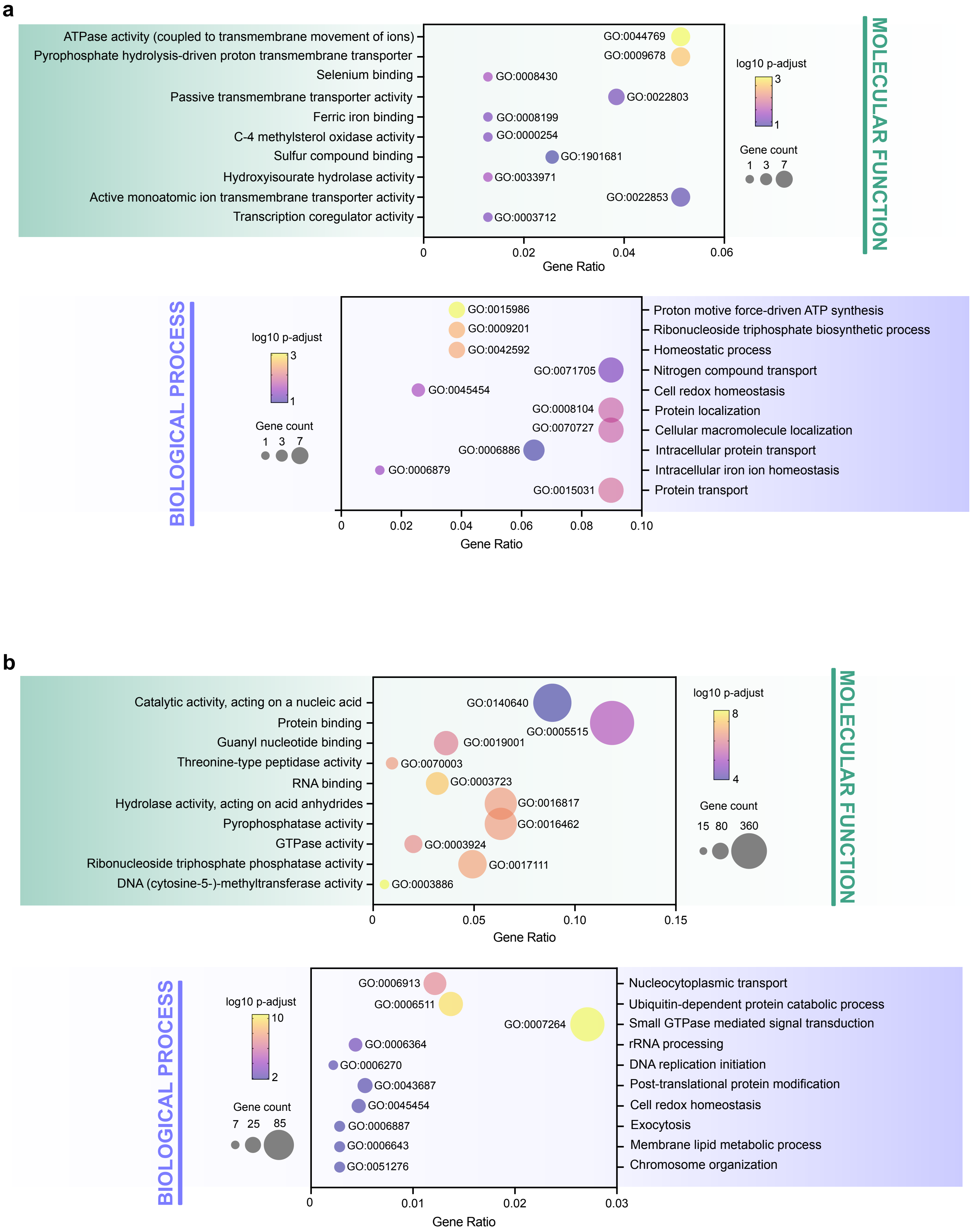
a
ATPase activity (coupled to transmembrane movement of ions)
Pyrophosphate hydrolysis-driven proton transmembrane transporter
Selenium binding
Passive transmembrane transporter activity
Ferric iron binding
C-4 methylsterol oxidase activity
Sulfur compound binding
Hydroxyisourate hydrolase activity
Active monoatomic ion transmembrane transporter activity
Transcription coregulator activity
GO:0044769
GO:0009678
GO:0008430
GO:0022803
GO:0008199
GO:0000254
GO:1901681
GO:0033971
GO:0022853
GO:0003712
0
0.02
0.04
0.06
Gene Ratio
log10 p-adjust
3
1
Gene count
1 3 7
MOLECULAR FUNCTION
BIOLOGICAL PROCESS
log10 p-adjust
3
1
Gene count
1 3 7
GO:0015986
GO:0009201
GO:0042592
GO:0071705
GO:0045454
GO:0008104
GO:0070727
GO:0006886
GO:0006879
GO:0015031
Proton motive force-driven ATP synthesis
Ribonucleoside triphosphate biosynthetic process
Homeostatic process
Nitrogen compound transport
Cell redox homeostasis
Protein localization
Cellular macromolecule localization
Intracellular protein transport
Intracellular iron ion homeostasis
Protein transport
0
0.02
0.04
0.06
0.08
0.10
Gene Ratio
b
Catalytic activity, acting on a nucleic acid
Protein binding
Guanyl nucleotide binding
Threonine-type peptidase activity
RNA binding
Hydrolase activity, acting on acid anhydrides
Pyrophosphatase activity
GTPase activity
Ribonucleoside triphosphate phosphatase activity
DNA (cytosine-5-)-methyltransferase activity
GO:0140640
GO:0005515
GO:0019001
GO:0070003
GO:0003723
GO:0016817
GO:0016462
GO:0003924
GO:0017111
GO:0003886
0
0.05
0.10
0.15
Gene Ratio
log10 p-adjust
8
4
Gene count
15 80 360
MOLECULAR FUNCTION
BIOLOGICAL PROCESS
log10 p-adjust
10
2
Gene count
7 25 85
GO:0006913
GO:0006511
GO:0007264
GO:0006364
GO:0006270
GO:0043687
GO:0045454
GO:0006887
GO:0006643
GO:0051276
Nucleocytoplasmic transport
Ubiquitin-dependent protein catabolic process
Small GTPase mediated signal transduction
rRNA processing
DNA replication initiation
Post-translational protein modification
Cell redox homeostasis
Exocytosis
Membrane lipid metabolic process
Chromosome organization
0
0.01
0.02
0.03
Gene Ratio

Supplementary Figure 5. GO enrichment analysis of 6mA methylated genes.

GO analysis of methylated genes of *Mucor* (A) and *Phycomyce*s (B). The top 400 genes more densely methylated (6mA) were considered for *Mucor* analysis and genes harboring a MAC in the range of -150bp and +400bp upstream and downstream the TSS, respectively, were considered for *Phycomyces*. The top 10 enriched terms of Molecular Function and Biological Process categories are indicated for each species.

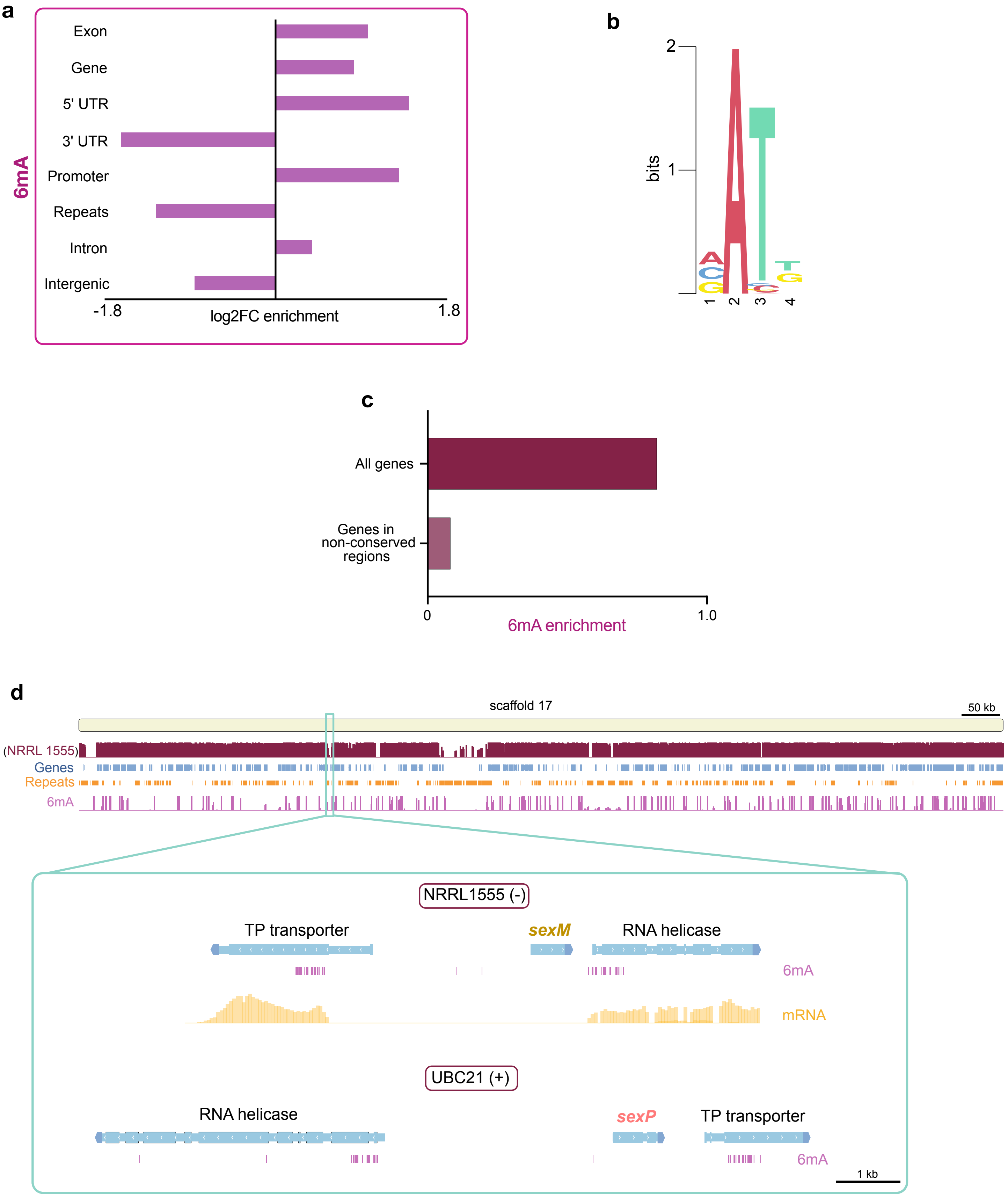

a
6mA
Exon
Gene
5' UTR
3' UTR
Promoter
Repeats
Intron
Intergenic
-1.8
log2FC enrichment
1.8
b
bits
2
1
1
2
3
4
c
All genes
Genes in non-conserved regions
0
6mA enrichment
1.0
d
scaffold 17
50 kb
(NRRL 1555)
Genes
Repeats
6mA
NRRL1555 (-)
TP transporter
sexM
RNA helicase
6mA
mRNA
UBC21 (+)
RNA helicase
sexP
TP transporter
6mA
1 kb

Supplementary Figure 6. 6mA in *P. blakesleeanus* UBC21.

(A) 6mA enrichment by genomic features (B) 6mA motif (VATB) for methylated sites in *P. blakesleeanus* UBC21. (C) Methylation (6mA) enrichment over genes located in variable genomic regions (non-conserved, n= 18) and in all genes (n=12840) between *P. blakesleeanus* NRRL1555 and *P. blakesleeanus* UBC21. A lower 6mA enrichment is observed compared to the complete set of genes. (D) View of the sex *locus* of *P. blakesleeanus* UBC21. Below is a detailed view of the 6mA clusters that were detected over the triose phosphate transporter and the RNA helicase genes on both *P. blakesleeanus* UBC21 and *P. blakesleeanus* NRRL1555 sex *loci*.

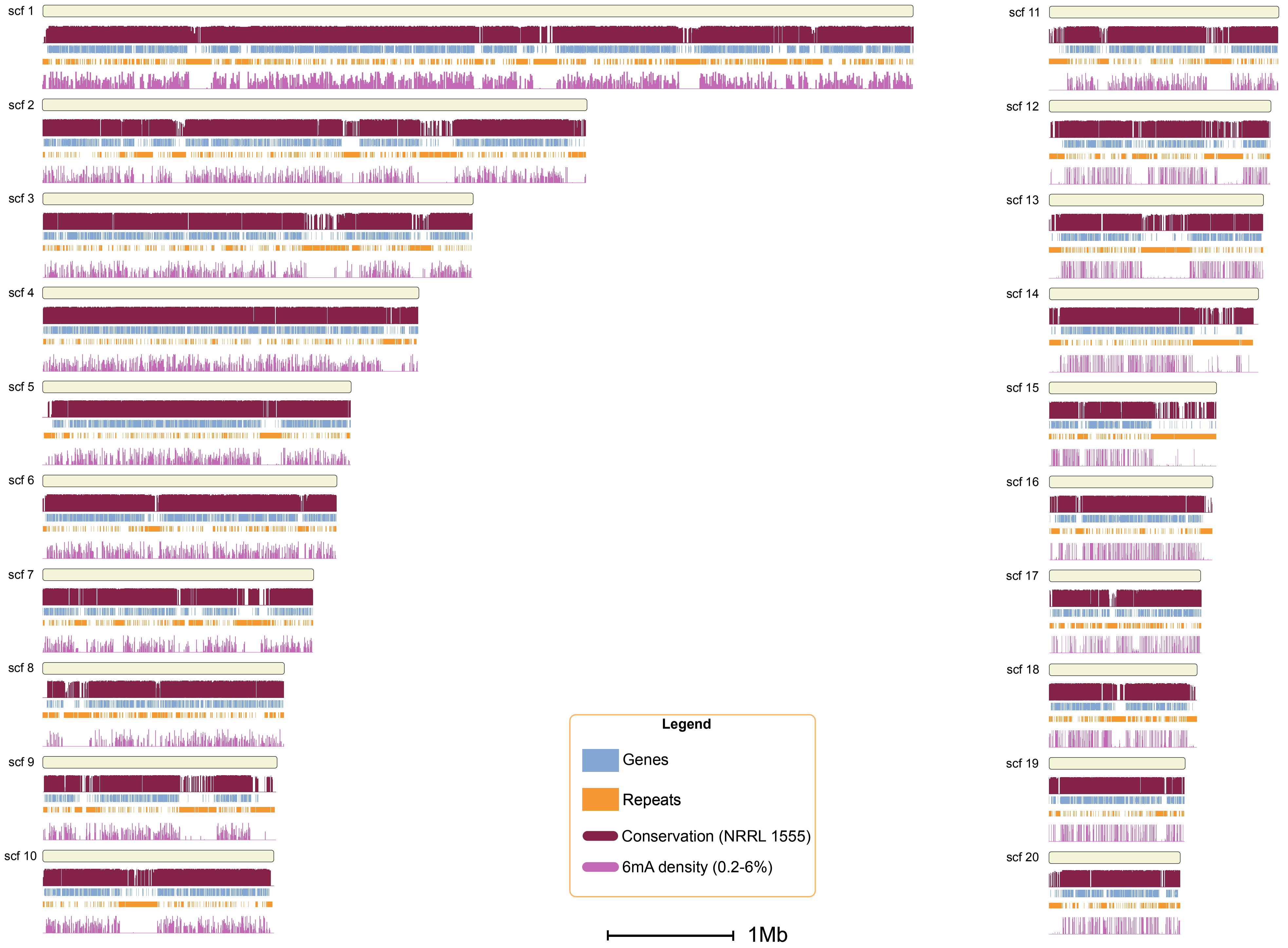
scf 1
scf 2
scf 3
scf 4
scf 5
scf 6
scf 7
scf 8
scf 9
scf 10
scf 11
scf 12
scf 13
scf 14
scf 15
scf 16
scf 17
scf 18
scf 19
scf 20
Legend
Genes
Repeats
Conservation (NRRL 1555)
6mA density (0.2-6%)
1Mb

Supplementary Figure 7. Distribution of 6mA in *P. blakesleeanus* UBC21.

P. blakesleeanus UBC21 scaffolds 1-20 indicating the nucleotide conservation level with *P. blakesleeanus* NRRL1555 (maroon track), gene density (blue track), repeats density (orange track), and 6mA (pink track).

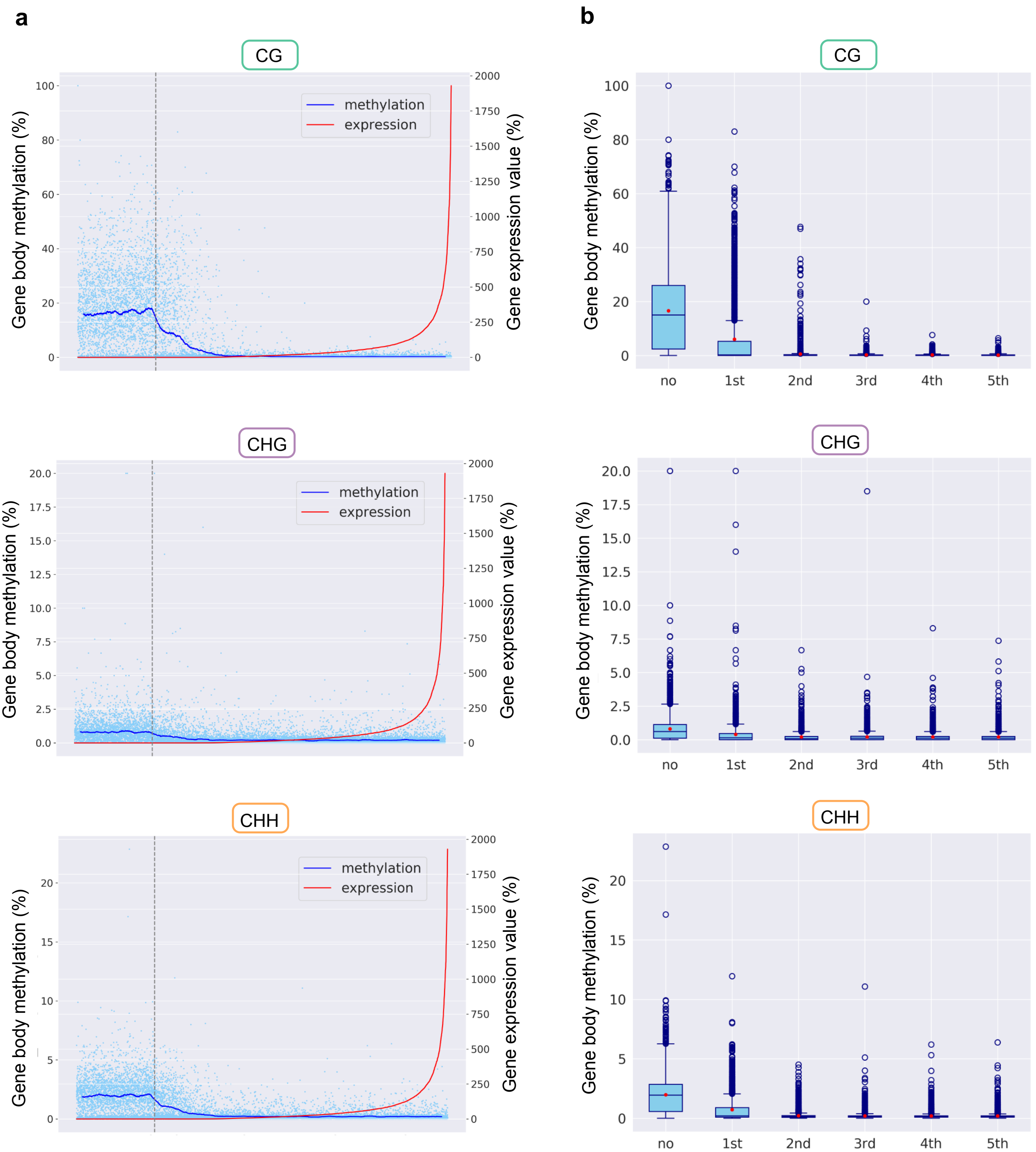

a
b
CG
CHG
CHH
methylation
expression
Gene body methylation (%)
Gene expression value (%)
no
1st
2nd
3rd
4th
5th

Supplementary Figure 8. 5mC and gene expression in the *Phycomyces* genome.

(A) Smooth graph diagrams of CG, CHG and CHH methylation (gene bodies) and expression levels for each gene. (B) Boxplots of genes ranked in six groups according to their expression levels (no = no expressed, 1 = low expression, 5 = high expression). The methylation level at gene bodies for each context is shown.

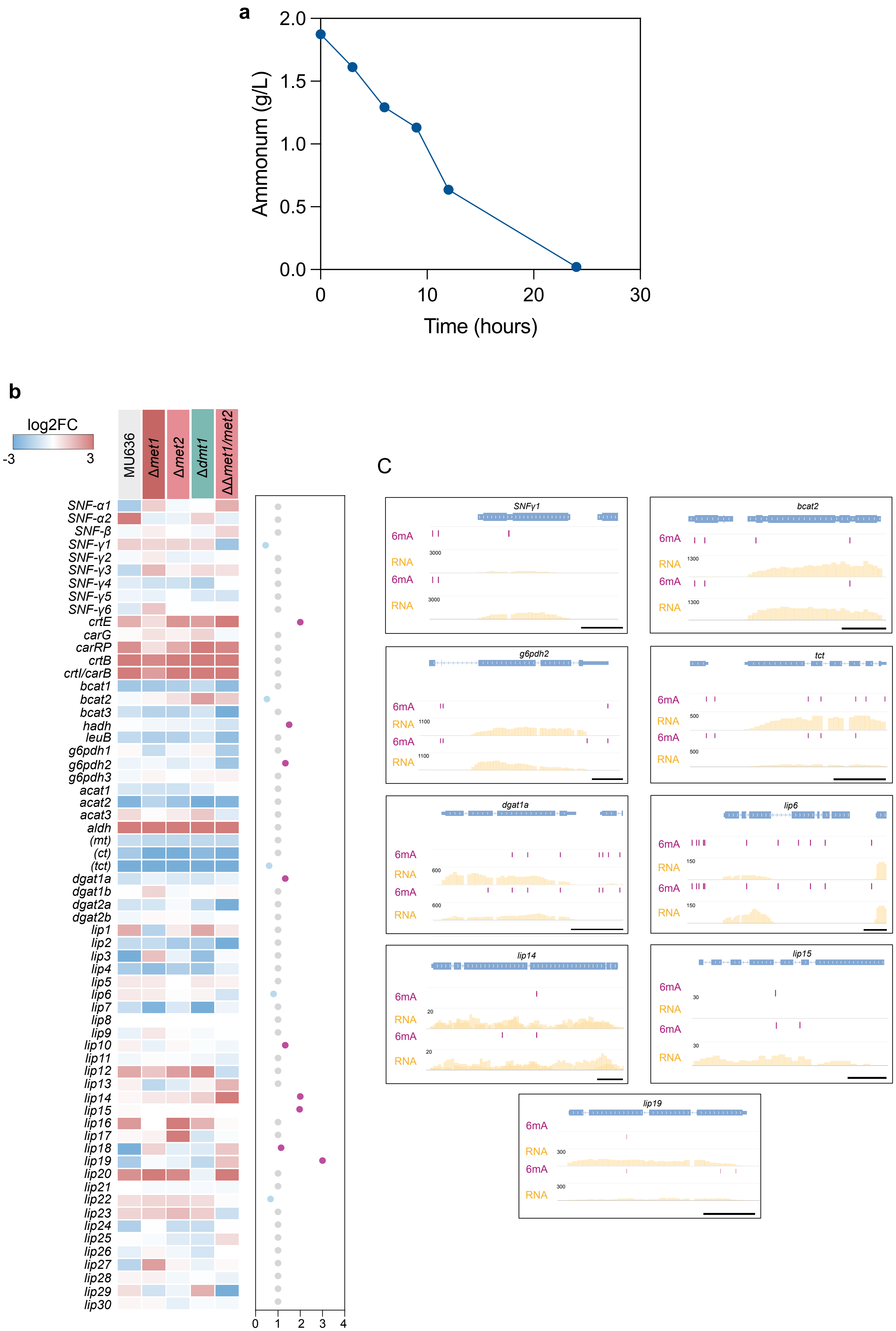
a
Ammonum (g/L)
Time (hours)
b
log2FC
-3
3
MU636
Δmet1
Δmet2
Δdmt1
ΔΔmet1/met2
SNF-α1
SNF-α2
SNF-β
SNF-γ1
SNF-γ2
SNF-γ3
SNF-γ4
SNF-γ5
SNF-γ6
crtE
carG
carRP
crtB
crtI/carB
bcat1
bcat2
bcat3
hadh
leuB
g6pdh1
g6pdh2
g6pdh3
acat1
acat2
acat3
aldh
(mt)
(ct)
(tct)
dgat1a
dgat1b
dgat2a
dgat2b
lip1
lip2
lip3
lip4
lip5
lip6
lip7
lip8
lip9
lip10
lip11
lip12
lip13
lip14
lip15
lip16
lip17
lip18
lip19
lip20
lip21
lip22
lip23
lip24
lip25
lip26
lip27
lip28
lip29
lip30
C
SNFγ1
bcat2
g6pdh2
tct
dgat1a
lip6
lip14
lip15
lip19
6mA
RNA

Supplementary Figure 9: (A) Nitrogen consumption in *M. lusitanicus* measured at 3, 6, 9, 12 and 24 hours (B) Log2FC of genes reported to be involved in lipid metabolism in *M. lusitanicus* in nitrogen-rich media (reduced lipid production) and in nitrogen-depleted media (increased lipid production). Genes more expressed in nitrogen-depleted are considered upregulated. Expression data of the wild-type strain (MU636) and the *met* and *dmt1* mutants were included. At the right, the methylation ratio for all genes analyzed is indicated for the MU636 strains (Nd/N). A ratio >1 indicates more methylated sites in treatment vs control and are indicated in red. A ratio <1 indicates lower methylated sites in treatment vs control and are indicated in blue. (C) Snapshots of the genes with a methylation ratio higher or lower than 1. Methylation sites are colored in purple, and mRNA tracks are colored in yellow for MU636 growing in nitrogen-rich media (above 6mA and mRNA tracks) and nitrogen-depleted media (below 6mA and mRNA tracks). Scale bar = 500 bp.

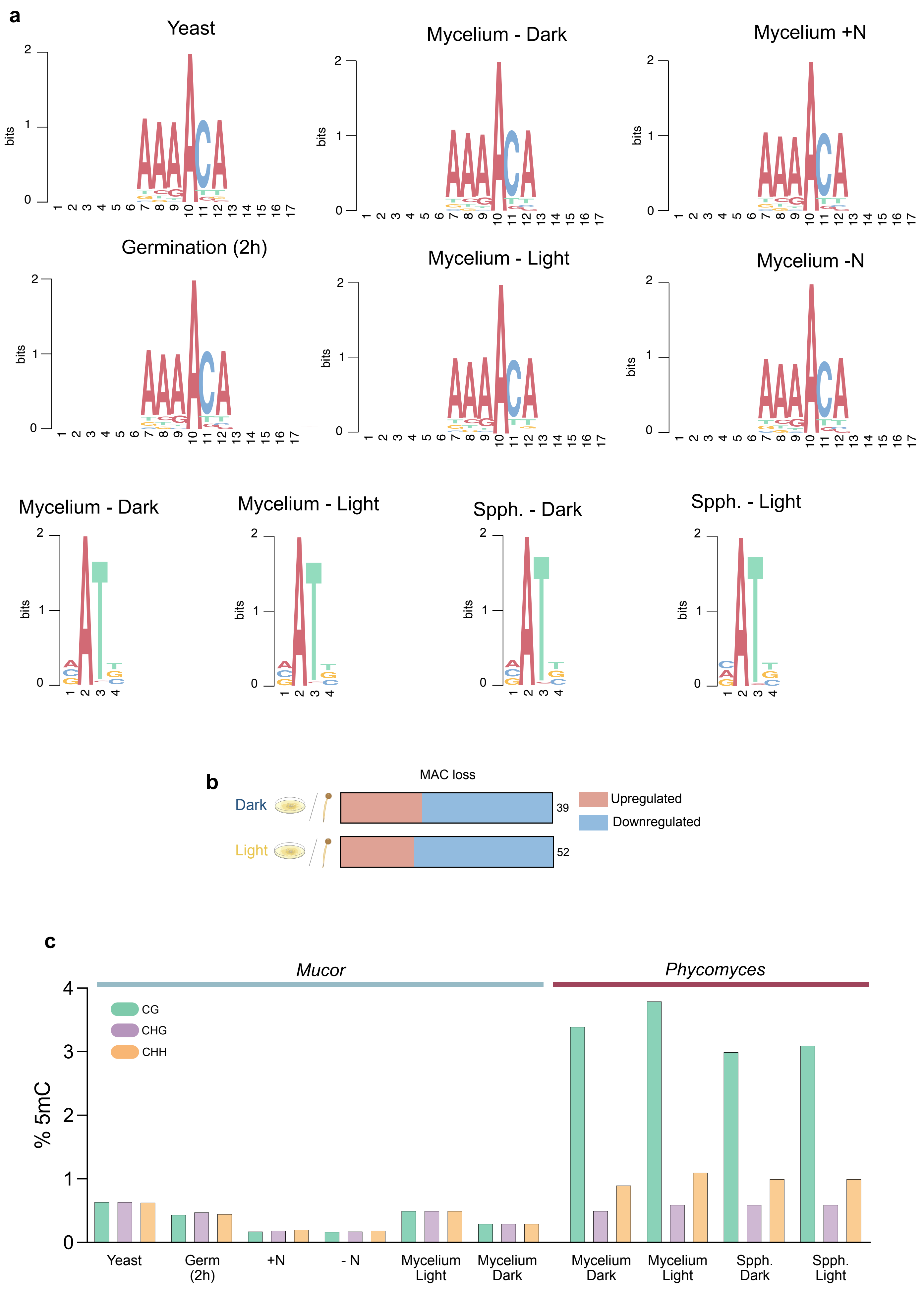
a
Yeast
Mycelium - Dark
Mycelium +N
Germination (2h)
Mycelium - Light
Mycelium -N
Mycelium - Dark
Mycelium - Light
Spph. - Dark
Spph. - Light
bits
b
MAC loss
Dark
Light
39
52
Upregulated
Downregulated
c
Mucor
Phycomyces
CG
CHG
CHH
% 5mC
Yeast
Germ
(2h)
+N
- N
Mycelium
Light
Mycelium
Dark
Mycelium
Dark
Mycelium
Light
Spph.
Dark
Spph.
Light

Supplementary Figure 10: (A) 6mA motifs for all wild-type samples sequenced in this work for both *Mucor* (top) and *Phycomyces* (bottom). (B) Proportion of upregulated (red) and downregulated (blue) genes among the differentially expressed genes that have lost a MAC. (C) Weighted 5mC levels indicated for each context for all samples sequenced from *Mucor* and *Phycomyces*. (Spph. = Sporangiophore).

a

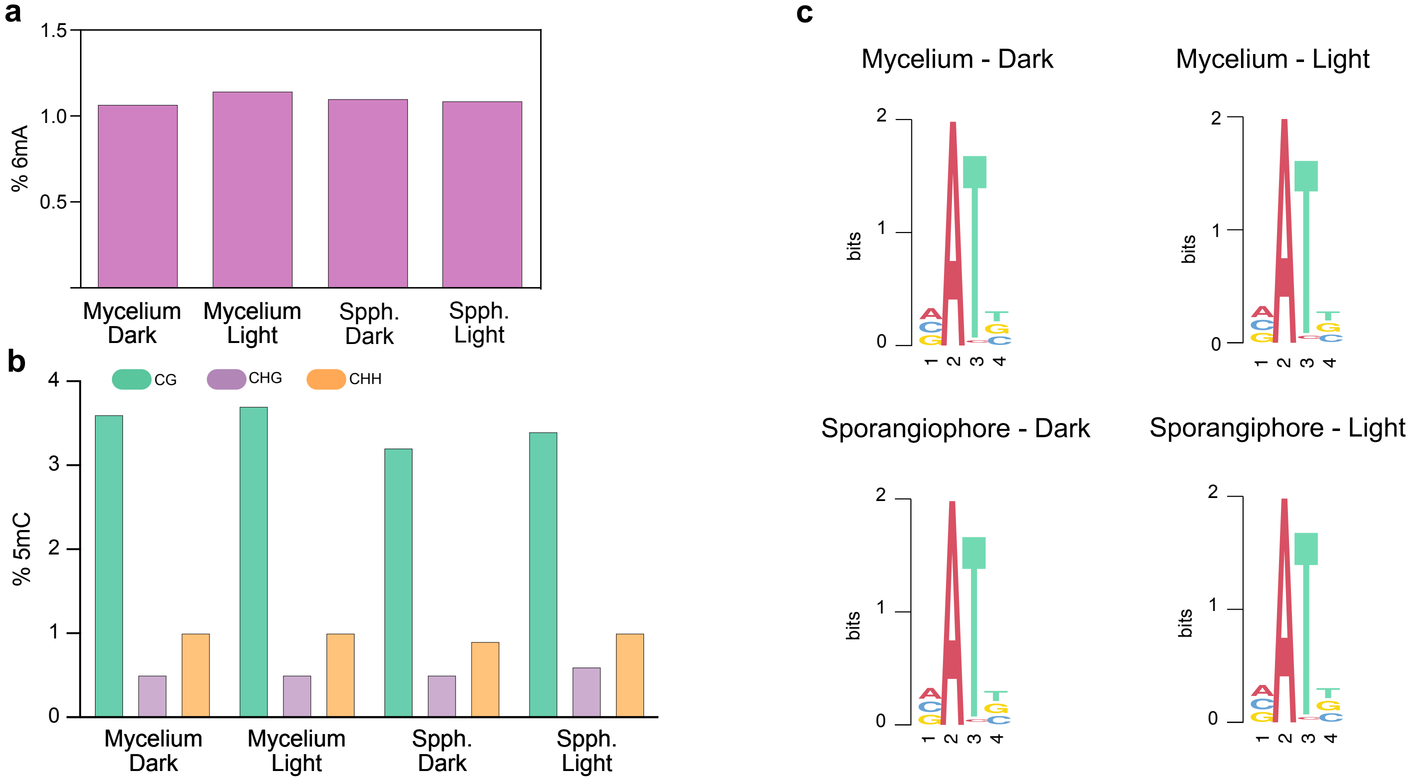

d

	Mycelium Light / Mycelium Dark	Sporangiophore Dark / Mycelium Dark	Sporangiophore Light / Sporangiophore Dark	Sporangiophore Light / Mycelium Light
Up-	0	2432	0	2620
Down-	0	2304	0	2396

e

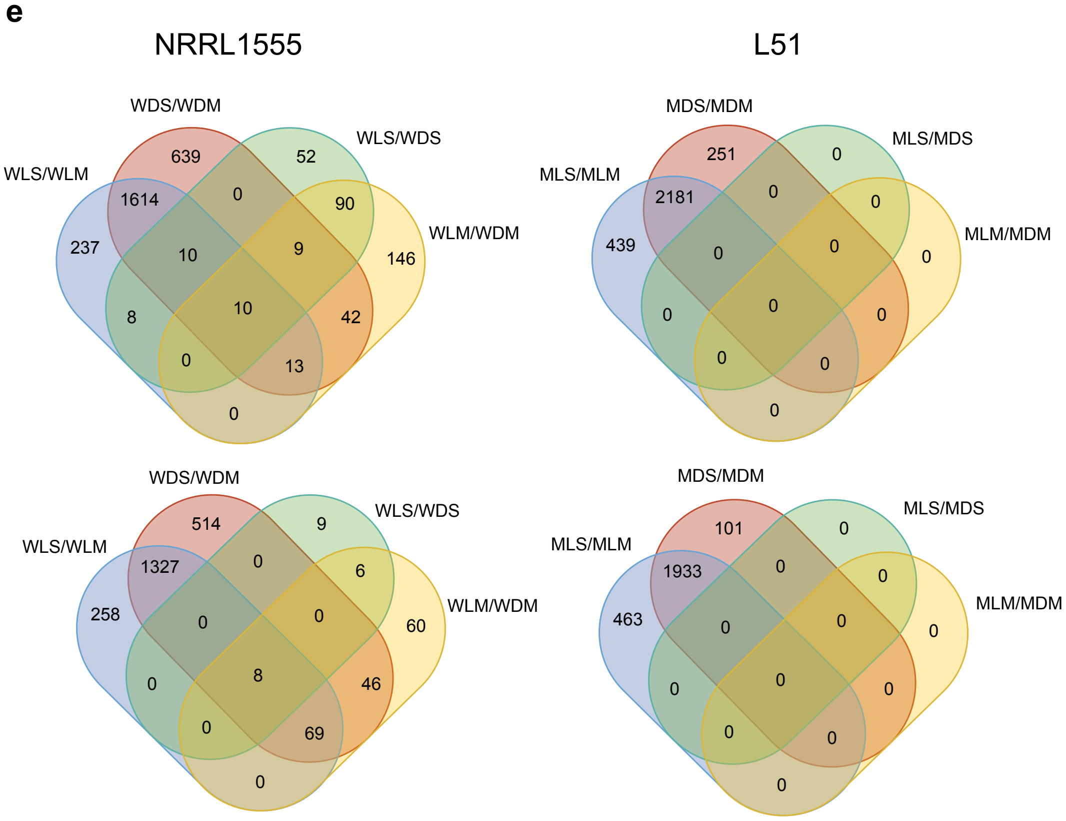

Supplementary Figure 11: (A) Detected 6mA levels for *P. blakesleeanus* L51 in the four growth conditions tested. (B) Weighted methylation levels at CG, CHG and CHH contexts for each condition sequenced. (C) 6mA motifs for the methylation sites identified in the samples sequenced. (D) Number of upregulated and downregulated genes for each comparison indicated above (treatment/control). Noteworthy, no DE genes were detected in dark growth vs. light growth comparisons in mycelium or sporangiophore growth stages. (E) Venn diagrams indicating the upregulated (top) genes and downregulated genes (bottom) for each comparison in the NRRL1555 strain and in the L51 strain. (WDM: NRRL1555 Dark Mycelium, WLM: NRRL1555 Light Mycelium, WDS: NRRL1555 Dark Sporangiophore, WLS: NRRL1555 Light Sporangiophore, MDM: L51 Dark Mycelium, MLM: L51 Light Mycelium, MDS: L51 Dark Sporangiophore, MLS: L51 Light Sporangiophore).

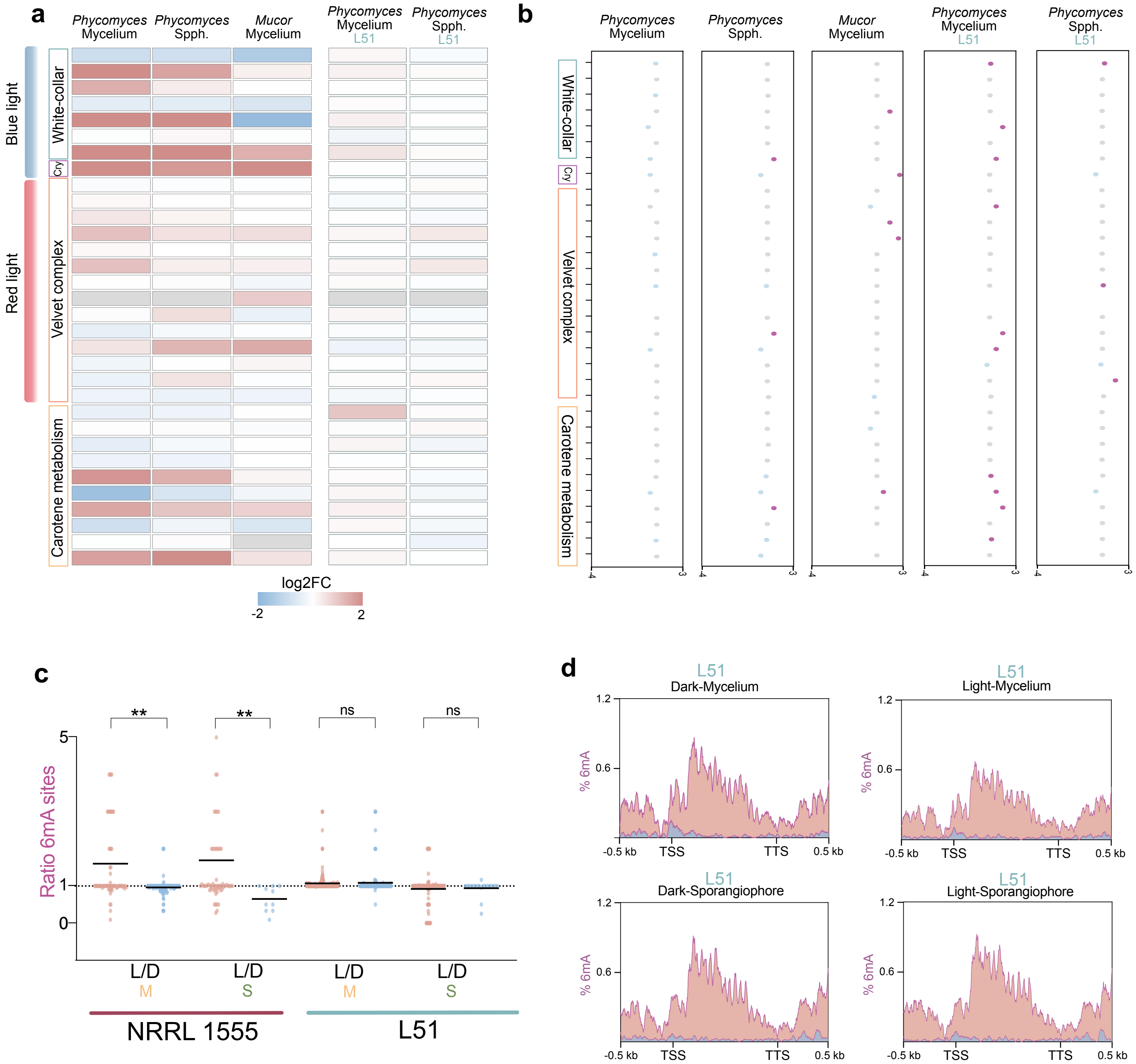
a
Phycomyces Mycelium
Phycomyces Spph.
Mucor Mycelium
Phycomyces Mycelium L51
Phycomyces Spph. L51
Blue light
Red light
White-collar
Cry
Velvet complex
Carotene metabolism
log2FC
-2
2
b
Phycomyces Mycelium
Phycomyces Spph.
Mucor Mycelium
Phycomyces Mycelium L51
Phycomyces Spph. L51
c
Ratio 6mA sites
**
ns
L/D
M
S
NRRL 1555
L51
d
L51
Dark-Mycelium
Light-Mycelium
Dark-Sporangiophore
Light-Sporangiophore
% 6mA
-0.5 kb
TSS
TTS
0.5 kb

Supplementary Figure 12: (A) log2FC of light response involved genes in light vs dark growth conditions for *Mucor* and *Phycomyces* mycelium and sporangiophore. The same is indicated for the *Phycomyces* blind mutant L51 strain. (B) Methylation ratio for all genes considered in panel A. A ratio >1 indicates more methylated sites in light vs dark and are indicated in red. A ratio <1 indicates lower methylated sites in light vs dark and are indicated in blue. **(**C) 6mA ratio of upregulated genes in the light response for each comparison in the NRRL1555 strain and the L51 strain. A Welch's (two-sided) test was performed for each NRRL1555 vs L51 comparison NRRL1555 P-val Mycelium L/D = 0.0017 , P-val Sporangiophore L/D = 0.0014, L51 P-val Mycelium L/D = ns , P-val Sporangiophore L/D = ns. ns = not significant). (D) 6mA distribution over the top 400 more highly expressed genes (red) and lower expressed genes (blue).

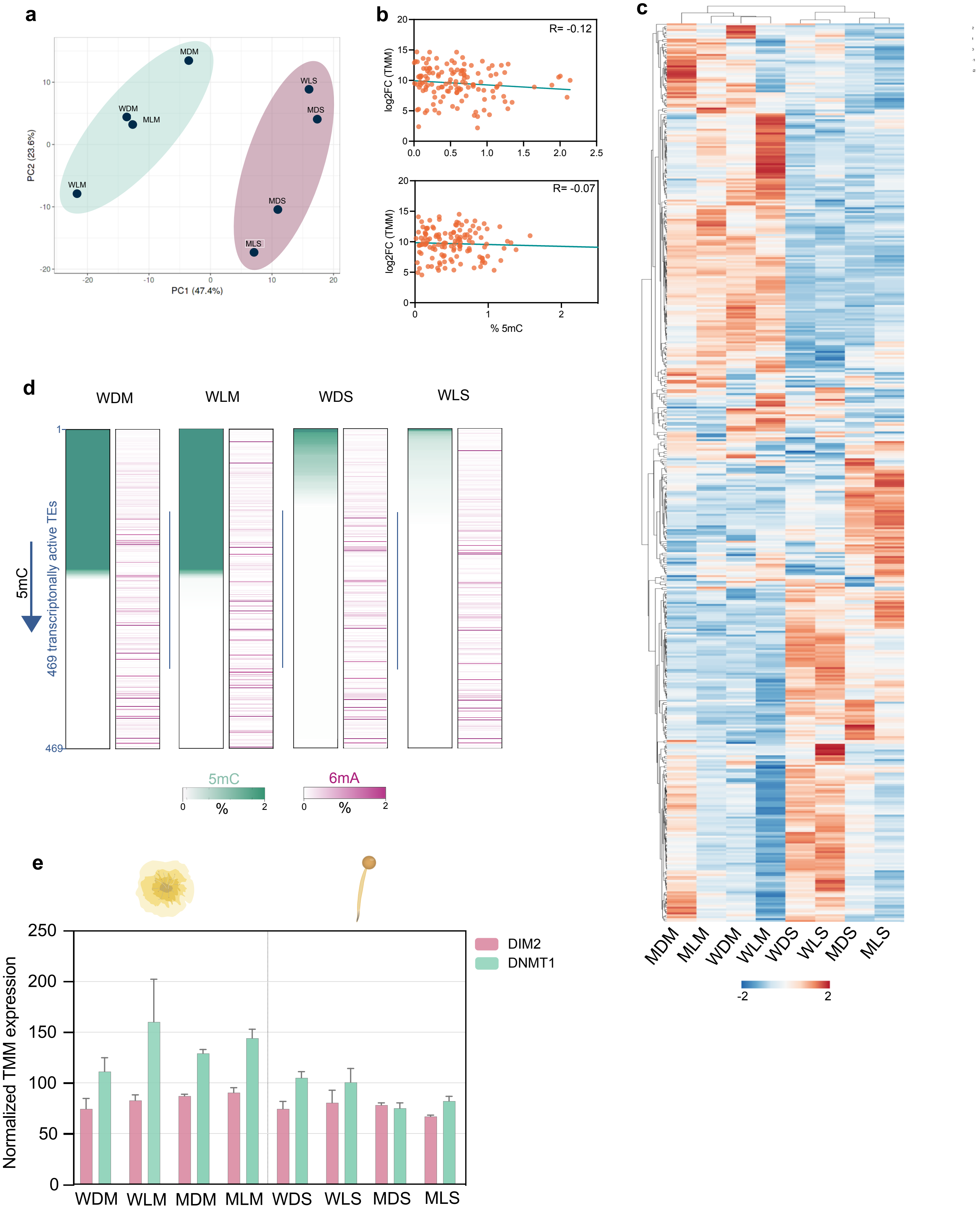

a
PC2 (23.6%)
PC1 (47.4%)
MDM
WDM
MLM
WLM
WLS
MDS
MDS
MLS
b
R= -0.12
log2FC (TMM)
R= -0.07
log2FC (TMM)
% 5mC
c
MDM
MLM
WDM
WLM
WDS
WLS
MDS
MLS
-2
2
d
WDM
WLM
WDS
WLS
5mC
469 transcriptonally active TEs
1
469
5mC
%
6mA
%
e
Normalized TMM expression
DIM2
DNMT1
WDM
WLM
MDM
MLM
WDS
WLS
MDS
MLS

Supplementary Figure 13. (A) Principal component analysis (PCA) of the expression patterns of transcriptionally active TE for all growth conditions analyzed (NRRL1555 dark-mycelium (WDM), light-mycelium (WLM), dark-sporangiophore (WDS) and light-sporangiophore (WLS), L51 dark-mycelium (MDM), light-mycelium (MLM), dark-sporangiophore (MDS) and light-sporangiophore (MDS)). (B) log2FC TMM for the 460 transcriptionally active TE and the average 5mC density across all copies of the same TE for WDM (top) and WDS (bottom). Pearson's correlation coefficient was computed for each comparison (top right corner of each plot). (C) Heatmap of transcriptionally active TE generated from normalized TMMs for each condition and hierarchically clustered (distance: correlation, clustering method: average). (D) Average methylation level (5mC and 6mA) for the 469 transcriptionally active transposons sorted in descending order according to their 5mC methylation level for each growth condition ((WDM), light-mycelium (WLM), dark-sporangiophore (WDS) and light-sporangiophore (WLS)). (E) Average TMM levels for DIM2, DNMT1, and DNMT2 methyltransferases in the growth stages and conditions assayed. Average values were calculated from three different biological replicates for each growth condition. Data is represented as mean +/- SD.

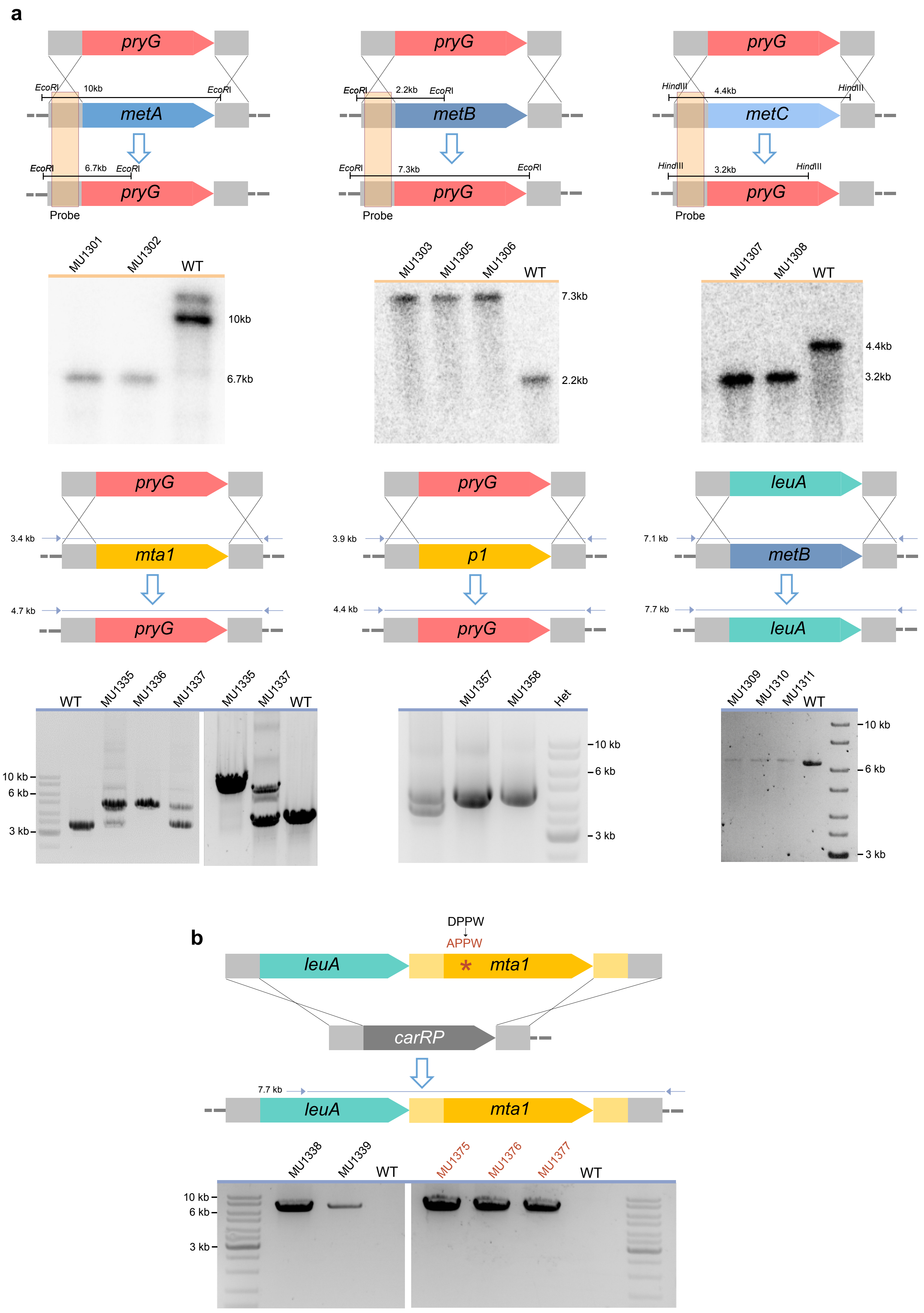

a
pryG
metA
metB
metC
EcoRI
10kb
6.7kb
2.2kb
7.3kb
HindIII
4.4kb
3.2kb
Probe
MU1301
MU1302
WT
MU1303
MU1305
MU1306
MU1307
MU1308
mta1
p1
leuA
3.4 kb
4.7 kb
3.9 kb
4.4 kb
7.1 kb
7.7 kb
MU1335
MU1336
MU1337
MU1357
MU1358
Het
MU1309
MU1310
MU1311
10 kb
6 kb
3 kb
b
DPPW
APPW
carRP
MU1338
MU1339
MU1375
MU1376
MU1377

Supplementary Figure 14 (A) Diagrams indicating the double homologous recombination events for the knockout of *metA*, *metB*, *metC*, *mta1* and *p1* genes. Southern blot validation with the probe and enzymes used for each case is indicated for *metA*, *metB* and *metC* mutant strains. PCR validation is indicated for *mta*1 and *p1* mutant strains. The name of each strain and the expected sizes for either mutant or WT nuclei are indicated on each panel. (B) Construct designs and schematic representations of genetic complementation of mta1 with the wild-type and mutated version of the protein. The results for PCR validation of the transformants are indicated below. The name of each strain and the expected sizes for either mutant or WT nuclei are indicated on each panel.

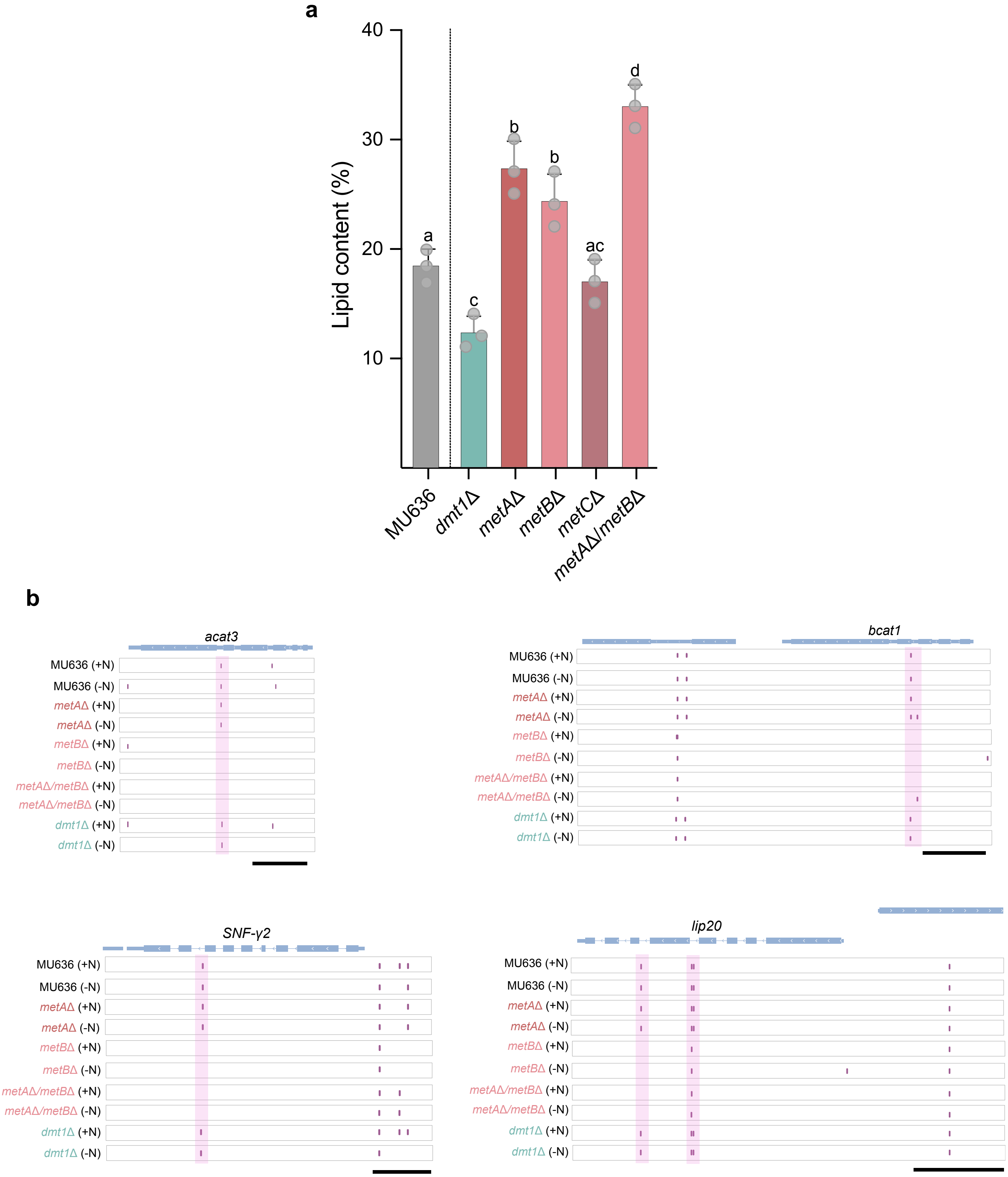
a
Lipid content (%)
40
30
20
10
a
c
b
b
ac
d
MU636
dmt1Δ
metAΔ
metBΔ
metCΔ
metAΔ/metBΔ
b
acat3
bcat1
SNF-γ2
lip20
MU636 (+N)
MU636 (-N)
metAΔ (+N)
metAΔ (-N)
metBΔ (+N)
metBΔ (-N)
metAΔ/metBΔ (+N)
metAΔ/metBΔ (-N)
dmt1Δ (+N)
dmt1Δ (-N)

Supplementary Figure 15. (A) Total lipid content (normalized to dry mass) of MU636 strain, *metA*Δ, *metB*Δ, *metC*Δ, *dmt1*Δ and *metA*Δ/*metB*Δ. Different letters indicate statistically significant differences, while the identical letters denote no significant differences, calculated using one-way ANOVA ($p < 0.001$, Tukey test). Mean ± SD was calculated from three biological replicates for each strain. (B) Snapshot of genes involved in the lipid metabolism that display a different expression pattern between the wild-type and mutant strains. 6mA sites are indicated for each strain in nitrogen-rich and nitrogen-depleted media. Sites lost in *metB* and double mutant *metA*/*metB* are indicated with pink boxes. Scale bar = 500bp.

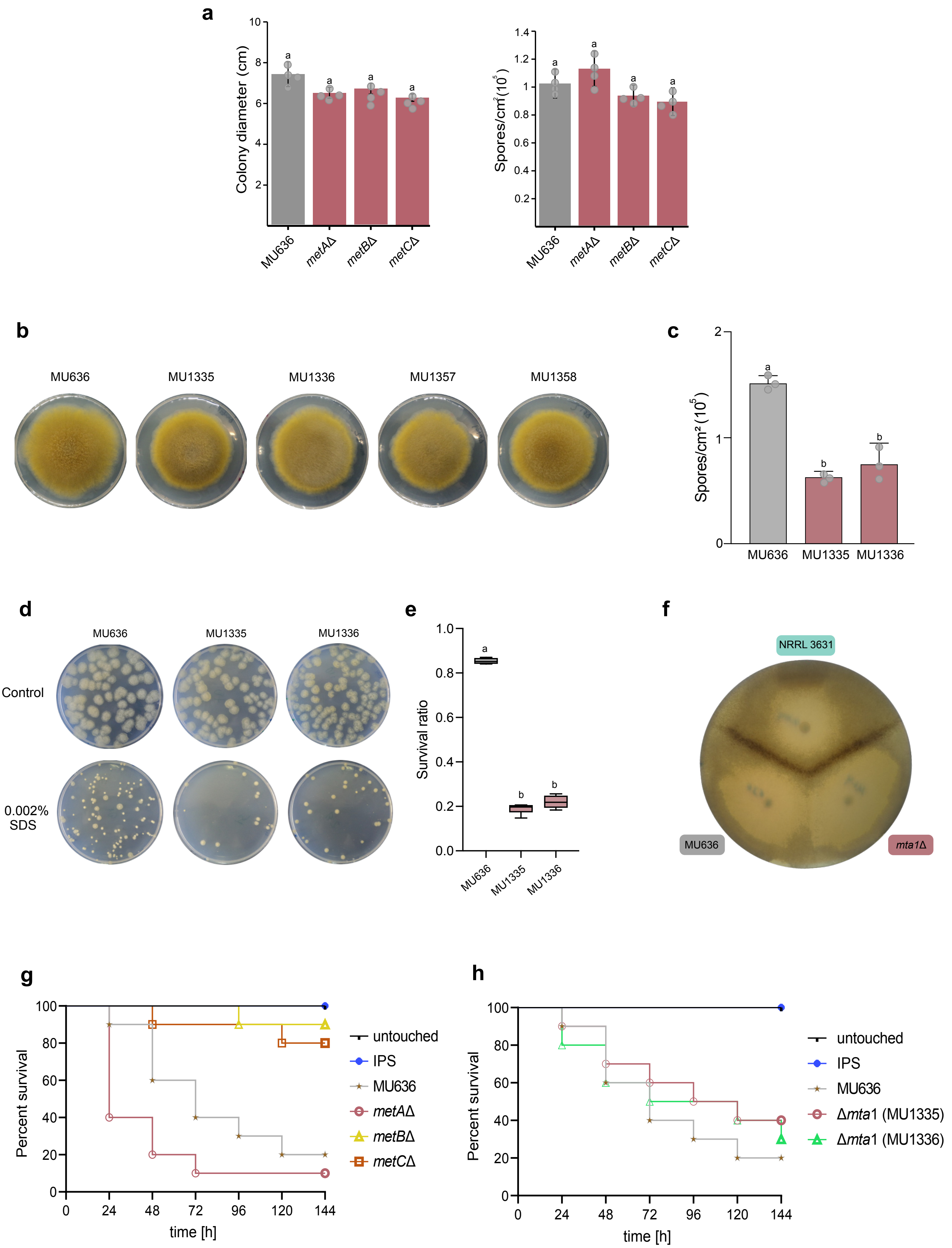
a
Colony diameter (cm)
MU636
metAΔ
metBΔ
metCΔ
Spores/cm² (10⁵)
b
MU636
MU1335
MU1336
MU1357
MU1358
c
Spores/cm² (10⁵)
MU636
MU1335
MU1336
d
MU636
MU1335
MU1336
Control
0.002% SDS
e
Survival ratio
MU636
MU1335
MU1336
f
NRRL 3631
MU636
mta1Δ
g
Percent survival
time [h]
untouched
IPS
MU636
metAΔ
metBΔ
metCΔ
h
Percent survival
time [h]
untouched
IPS
MU636
Δmta1 (MU1335)
Δmta1 (MU1336)

Supplementary Figure 16. (A) Colony diameter (cm) and sporulation quantification for MU636, *metA*Δ, *metB*Δ *and metC*Δ. Different letters indicate statistically significant differences, while the identical letters denote no significant differences, calculated using one-way ANOVA ($p < 0.001$,Tukey test). Data is represented as mean +- SD. Four measures were taken, one for each biological replicate. (B) Growth of two *mta1*Δ mutant strains (MU1335 and MU1336) and two *p1*Δ mutant strains (MU1557 and MU1358) compared to the wild-type strain MU636. (C) Spore production of MU636 and two independent *mta1*Δ mutant strains. Statistical differences were determined as described in (A). Data is represented as mean +/- SD. Three biological replicates were measured for each strain. (D) Growth of MU636 and two independent *mta1*Δ mutant strains in media with and without 0.002% SDS. (E) Number of colonies in control and SDS plates was used to calculate the survival ratio to SDS for each strain tested. Statistical differences were determined as described in (A). (F) Production of zygospores as a result of sexual reproduction between complementary mating types, plus (+) for NRRL 3631 and minus (-) for MU636 and the *mta1*Δ mutant strain MU1335. No obvious differences were detected for MU636 and MU1335 in their ability to mate with NRRL 3631. (G) and (H) survival curves for G. mellonella larvae inoculated with methyltransferase mutants. The survival rate was compared with the untouched and IPS-injected larvae with MantelCox test (p-value <0.0001)

Supplementary Table 1. Fungal species included in 6mA detection analyses

Species	Genome Size (Mb)	Reference	DOI	Project PI	Proposal ID	Accession	% 6mA
Absidia padenii NRRL 2977	34.33	2	https://doi.org/10.46936/10.25585/60001062	Joseph Spatafora	1978	https://mycocosm.jgi.doe.gov/Chlpad1	2,84
Absidia repens	47.42	4	https://doi.org/10.46936/10.25585/60001019	Joseph Spatafora	662	http://mycocosm.jgi.doe.gov/Absrep1	1,59
Anaeromyces robustus	71.69	4	https://doi.org/10.46936/jejc.proj.2013.48080/60005295	Michelle O'Malley	1373	http://mycocosm.jgi.doe.gov/Anasp1	1,71
Basidiobolus meristosporus	89.49	4	https://doi.org/10.46936/10.25585/60001019	Andrii Gryganskyi	662	http://mycocosm.jgi.doe.gov/Basme2finSC	1,6
Blakeslea trispora NRRL 2456	37.51	2	https://doi.org/10.46936/10.25585/60001019	Joseph Spatafora	662	https://mycocosm.jgi.doe.gov/Blatri1	0,69
Catenaria anguillulae	41.34	4	https://doi.org/10.46936/10.25585/60001019	Igor Grigoriev	662	http://mycocosm.jgi.doe.gov/Catan2	0,02
Choanephora cucurbitarum NRRL 2744	35.11	2	https://doi.org/10.46936/10.25585/60001062	Jason E. Stajich	1978	https://mycocosm.jgi.doe.gov/Chocucu1	0,83
Chytridium lagenaria Arg66	42.38	1	https://doi.org/10.46936/10.25585/60001062	Joseph Spatafora	1978	https://mycocosm.jgi.doe.gov/Chylag1	ND
Chytriomyces hyalinus JEL632	38.10	1	https://doi.org/10.46936/10.25585/60001019	Timothy Y. James	662	https://mycocosm.jgi.doe.gov/Chyhya1	0,12
Circinella umbellata NRRL 1351	51.27	2	https://doi.org/10.46936/10.25585/60001019	Joseph Spatafora	662	https://mycocosm.jgi.doe.gov/Cirumb1	1,21
Cladochytrium polystomum WB228	81.19	1	https://doi.org/10.46936/10.25585/60001062	Joseph Spatafora	1978	https://mycocosm.jgi.doe.gov/Clapol1_1	ND
Cladochytrium replicatum JEL 714	50.67	1	https://doi.org/10.46936/10.25585/60001019	Timothy Y. James	662	https://mycocosm.jgi.doe.gov/Clarep1_1	0,21
Clohesyomyces aquaticus	49.68	4	https://doi.org/10.46936/10.25585/60001019	Jon Karl Magnuson	662	http://mycocosm.jgi.doe.gov/Cloaq1	0,04
Coemansia mojavensis RSA 71	14.29	2	https://doi.org/10.46936/10.25585/60001062	Joseph Spatafora	1978	https://mycocosm.jgi.doe.gov/Coemoj1	0,98
Coemansia spiralis RSA 1278	18.93	2	https://doi.org/10.46936/10.25585/60001062	Joseph Spatafora	1978	https://mycocosm.jgi.doe.gov/Coespi1	1,24
Cokeromyces recurvatus NRRL 2243	28.12	2	https://doi.org/10.46936/10.25585/60001062	Joseph Spatafora	1978	https://mycocosm.jgi.doe.gov/Cokrec1	2,71
Dichotomocladium elegans RSA 919-	39.78	2	https://doi.org/10.46936/10.25585/60001062	Joseph Spatafora	1978	https://mycocosm.jgi.doe.gov/Dicele1	2,19
Entophlyctis helioformis JEL805	30.92	1	https://doi.org/10.46936/10.25585/60001062	Joseph Spatafora	1978	https://mycocosm.jgi.doe.gov/Enthel1	0,12
Fennellomyces sp. ATCC 46495	45.90	3	https://doi.org/10.46936/10.25585/60001062	Joseph Spatafora	1978	https://mycocosm.jgi.doe.gov/Fenlin1	1,55
Fimicolochytrium jonesii JEL569	30.62	1	https://doi.org/10.46936/10.25585/60001062	Timothy Y. James	1978	https://mycocosm.jgi.doe.gov/Fimjon1	1,84
Gaertneriomyces semiglobifer Barr 43	20.92	1	https://doi.org/10.46936/10.25585/60001062	Joseph Spatafora	1978	https://mycocosm.jgi.doe.gov/Gaesem1	ND
Gamsiella multidivaricata RSA 2152	37.67	2	https://doi.org/10.46936/10.25585/60001062	Joseph Spatafora	1978	https://mycocosm.jgi.doe.gov/Mormul1	1,27
Gilbertella persicaria var. persicaria CBS 190.32	25.73	2	https://doi.org/10.46936/10.25585/60001062	Joseph Spatafora	1978	https://mycocosm.jgi.doe.gov/Gilper1	1,4
Gongronella butleri CBS 227.36+	33.01	2	https://doi.org/10.46936/10.25585/60001062	Joseph Spatafora	1978	https://mycocosm.jgi.doe.gov/Gonbut1	1,05
Gorgonomyces haynaldii MP57	13.98	1	https://doi.org/10.46936/10.25585/60001062	Timothy Y. James	1978	https://mycocosm.jgi.doe.gov/Gorhay1	0,71
Halteromyces radiatus CBS 162.75	26.22	2	https://doi.org/10.46936/10.25585/60001062	Joseph Spatafora	1978	https://mycocosm.jgi.doe.gov/Halrad1	2,37
Hesseltinella vesiculosa	27.22	4	https://doi.org/10.46936/10.25585/60001019	Timothy Y. James	662	http://mycocosm.jgi.doe.gov/Hesve2finisherSC	2,8
Hyaloraphidium curvatum SAG235-1	31.93	1	https://doi.org/10.46936/10.25585/60001062	Timothy Y. James	1978	https://mycocosm.jgi.doe.gov/Hyacur1	0,21
Kickxella alabastrina RSA 675	22.26	2	https://doi.org/10.46936/10.25585/60001062	Joseph Spatafora	1978	https://mycocosm.jgi.doe.gov/Kicala1	0,81
Kockovaella imperatae	17.47	4	https://doi.org/10.46936/10.25585/60001019	Joseph Spatafora	662	http://mycocosm.jgi.doe.gov/Kocim1	0,04
Leucosporidiella creatinivora	26.33	4	https://doi.org/10.46936/10.25585/60001019	Jon Karl Magnuson	662	http://mycocosm.jgi.doe.gov/Leucr1	0,02
Linderina pennispora	26.20	4	https://doi.org/10.46936/10.25585/60001019	Timothy Y. James	662	http://mycocosm.jgi.doe.gov/Linpe1	0,31
Lobosporangium transversale	42.77	4	https://doi.org/10.46936/10.25585/60001019	Joseph Spatafora	662	http://mycocosm.jgi.doe.gov/Lobtra1	1,57
Mucor lusitanicus CBS 277.49	36,61	This Study	https://doi.org/10.46936/10.25585/60001127	Victoriano Garre	503418	https://mycocosm.jgi.doe.gov/Mucci3	0.5
Mucor mucedo NRRL 3635	46.09	3	https://doi.org/10.46936/10.25585/60001062	Jason E. Stajich	1978	https://mycocosm.jgi.doe.gov/Mucmuc1	1,42
Mycotypha africana NRRL 2978	29.20	2	https://doi.org/10.46936/10.25585/60001019	Joseph Spatafora	662	https://mycocosm.jgi.doe.gov/Mycafr1	1,88
Obelidium mucronatum JEL802	49.46	1	https://doi.org/10.46936/10.25585/60001019	Jason E. Stajich	662	https://mycocosm.jgi.doe.gov/Obemuc1	0,19
Phascolomyces articulosus RSA 2281	47.71	2	https://doi.org/10.46936/10.25585/60001062	Joseph Spatafora	1978	https://mycocosm.jgi.doe.gov/Phaart1	1,4
Phycomyces blakesleeanus L51	62.09	This Study	https://doi.org/10.46936/10.25585/60001127	Victoriano Garre	503418	https://mycocosm.jgi.doe.gov/Phybl_L51_1	1,15
Phycomyces blakesleeanus NRRL 1555	58,78	This Study	https://doi.org/10.46936/10.25585/60001127	Victoriano Garre	503418	https://mycocosm.jgi.doe.gov/Phybl4	1,13
Phycomyces blakesleeanus UBC21	56,67	This Study	https://doi.org/10.46936/10.25585/60001127	Victoriano Garre	503418	https://mycocosm.jgi.doe.gov/PhyblU21_2	1.04
Phycomyces nitens S608	37.68	2	https://doi.org/10.46936/10.25585/60001062	Joseph Spatafora	1978	https://mycocosm.jgi.doe.gov/PnitS608_1	1,46
Pilaira anomala RSA1997	34.90	2	https://doi.org/10.46936/10.25585/60001062	Joseph Spatafora	1978	https://mycocosm.jgi.doe.gov/Pilano1	1,33
Pilobolus umbonatus NRRL 6349	34.77	2	https://doi.org/10.46936/10.25585/60001019	Joseph Spatafora	662	https://mycocosm.jgi.doe.gov/Pilumb1	0,83
Piromyces finnis	56.46	4	https://doi.org/10.46936/jejc.proj.2013.48080/60005295	Michelle O'Malley	1373	http://mycocosm.jgi.doe.gov/Pirfi3	1,68
Polychytrium aggregatum JEL109	64.92	1	https://doi.org/10.46936/10.25585/60001019	Timothy Y. James	662	https://mycocosm.jgi.doe.gov/Polagg1_1	0,13
Powellomyces hirtus BR81	29.43	1	https://doi.org/10.46936/10.25585/60001062	Timothy Y. James	1978	https://mycocosm.jgi.doe.gov/Powhir1	1,43
Protomyces lactucaedebilis	12.93	4	https://doi.org/10.46936/10.25585/60001019	Jon Karl Magnuson	662	http://mycocosm.jgi.doe.gov/Prola1	0,1
Pseudomassariella vexata	44.71	4	https://doi.org/10.46936/10.25585/60001019	Jon Karl Magnuson	662	http://mycocosm.jgi.doe.gov/Pseve2	0,03
Radiomyces spectabilis NRRL 2753	30.41	3	https://doi.org/10.46936/10.25585/60001019	Timothy Y. James	662	https://mycocosm.jgi.doe.gov/Radspe1	2,45
Rhizoclosmatium globosum	57.02	4	https://doi.org/10.46936/10.25585/60001019	Jason E. Stajich	662	http://mycocosm.jgi.doe.gov/Rhihy1	0,05
Rhizophagus irregularis C2	159.8	5	https://doi.org/10.1038/s42003-021-02414-5	NA	NA	ENA: RJEB33553	0.2
Spinellus fusiger NRRL 22323	38.20	3	https://doi.org/10.46936/10.25585/60001019	Joseph Spatafora	662	https://mycocosm.jgi.doe.gov/Spifus1	0,94
Sporodiniella umbellata MES-1446	26.24	2	https://doi.org/10.46936/10.25585/60001062	Joseph Spatafora	1978	https://mycocosm.jgi.doe.gov/Spoumb1	1,66
Syncephalastrum racemosum	30.75	4	https://doi.org/10.46936/10.25585/60001019	Joseph Spatafora	662	http://mycocosm.jgi.doe.gov/Synrac1	1,47
Syncephalis fuscata S228	29.36	1	https://doi.org/10.46936/10.25585/60001019	Joseph Spatafora	662	https://mycocosm.jgi.doe.gov/Synfus1	1,07
Syncephalis plumigaleata NRRL S24	32.91	2	https://doi.org/10.46936/10.25585/60001019	Timothy Y. James	662	https://mycocosm.jgi.doe.gov/Synplu1	1,51
Thamnidium elegans NRRL 2467	34.25	2	https://doi.org/10.46936/10.25585/60001019	Joseph Spatafora	662	https://mycocosm.jgi.doe.gov/Thaele1	1,38
Tremella encephala	19.79	4	https://doi.org/10.46936/10.25585/60001019	Jon Karl Magnuson	662	http://mycocosm.jgi.doe.gov/Treen1	0,05
Triparticalcar arcticum BR59	31.55	1	https://doi.org/10.46936/10.25585/60001062	Timothy Y. James	1978	https://mycocosm.jgi.doe.gov/Triarc1	1,04
Umbelopsis sp. AD052	22.50	2	https://doi.org/10.46936/10.25585/60001062	Joseph Spatafora	1978	https://mycocosm.jgi.doe.gov/Umbsp_AD052_1	1,68
Zychaea mexicana RSA 1403	50.64	2	https://doi.org/10.46936/10.25585/60001062	Joseph Spatafora	1978	https://mycocosm.jgi.doe.gov/Zycmex1	1,65

Supplementary Table 2. Samples generated and sequenced in this work

Species	Growth Condition	6mA sites	% 6mA	% sym	MACs	% 6mA in MACs	% 5mC	%CG	%CHG	%CHH
Phycomyces blakesleeanus (NRRL1555)	Mycelium - Dark	386699	1.13	92.32	9703	93.4	1.12	3.4	0.5	0.9
Phycomyces blakesleeanus (NRRL1555)	Mycelium - Light	360738	1,05	94.76	9819	94.54	1.34	3.8	0.6	1.1
Phycomyces blakesleeanus (NRRL1555)	Sporangiophore - Dark	364922	1.06	93.03	9724	92.8	1.16	3	0.6	1
Phycomyces blakesleeanus (NRRL1555)	Sporangiophore - Light	353919	1.03	93.16	9767	92.9	1.23	3.1	0.6	1
Phycomyces blakesleeanus (L51)	Mycelium - Dark	356419	1.04	94.88	9774	94.6	1.20	3.6	0.5	1
Phycomyces blakesleeanus (L51)	Mycelium - Light	392729	1.14	91.72	9634	91.5	1.24	3.7	0.5	1
Phycomyces blakesleeanus (L51)	Sporangiophore - Dark	377866	1.10	92.4	9697	92.3	1.10	3.2	0.5	0.9
Phycomyces blakesleeanus (L51)	Sporangiophore - Light	373252	1.09	93.40	9715	93.2	1.19	3.4	0.6	1
Phycomyces blakesleeanus (UBC21)	Mycelium - Dark	386062	1.06	90.32	9248	90.1				
Mucor lusitanicus (CBS 277.49)	Mycelium - Dark	108435	0.51	0.31	200	0.17	0.31	0.3	0.3	0.3
Mucor lusitanicus (MU636)	Mycelium - Light	53497	0.25	0.95	73	0.69	0.54	0.5	0.5	0.5
*Mucor lusitanicus (*MU636)	Mycelium - Dark	49568	0.23	1.03	71	0.75	0.32	0.3	0.3	0.3
Mucor lusitanicus (MU636)	Yeast	53350	0.25	0.80	65	0.52	0.64	0.64	0.64	0.63
Mucor lusitanicus (MU636)	Mycelium (2h transition from yeast)	55445	0.26	0.80	64	0.51	0.45	0.44	0.48	0.45
Mucor lusitanicus (MU636)	Mycelium - Nitrogen rich	55909	0.26	0.80	63	0.53	0.19	0.18	0.19	0.20
Mucor lusitanicus (MU636)	Mycelium - Nitrogen depletion	62238	0.27	0.82	70	0.53	0.19	0.17	0.18	0.19
Mucor lusitanicus (MU1301)	Mycelium - Nitrogen rich	40124	0.19	0.55	31	0.26	0.15	0.14	0.15	0.15
Mucor lusitanicus (MU1301)	Mycelium - Nitrogen depletion	52278	0.25	0.60	48	0.33	0.15	0.14	0.15	0.15
Mucor lusitanicus (MU1306)	Mycelium - Light	16289	0.077	2.81	64	1.79	0.11	0.11	0.11	0.12
Mucor lusitanicus (MU1306)	Mycelium - Dark	6226	0,039	3.66	42	2.5	0.11	0.10	0.11	0.11
Mucor lusitanicus (MU1306)	Mycelium - Nitrogen rich	10454	0.049	2.70	37	1.44	0.11	0.10	0.10	0.11
Mucor lusitanicus (MU1306)	Mycelium - Nitrogen depletion	19655	0.093	2.09	60	1.30	0.11	0.10	0.11	0.11
Mucor lusitanicus (MU1310)	Mycelium - Nitrogen rich	12914	0.061	2.06	35	1.10	0.11	0.11	0.11	0.11
Mucor lusitanicus (MU1310)	Mycelium - Nitrogen depletion	13239	0.063	2.39	47	1.32	0.11	0.10	0.11	0.11
Mucor lusitanicus (MU1317)	Yeast	44250	0.21	0.91	59	0.57	0.14	0.13	0.14	0.14
Mucor lusitanicus (MU1317)	Mycelium (2h transition from yeast)	52673	0.24	0.87	67	0.52	0.12	0.11	0.12	0.12
Mucor lusitanicus (MU1317)	Mycelium - Nitrogen rich	61511	0.27	0.86	70	0.55	0.12	0.12	0.12	0.13
Mucor lusitanicus (MU1317)	Mycelium - Nitrogen depletion	47050	0.22	0.96	66	0.65	0.18	0.18	0.18	0.19
Mucor lusitanicus (MU1335)	Mycelium - Light	46589	0.22	0	0	0	0.60	0.59	0.60	0.59
*Mucor lusitanicus (*MU1357)	Mycelium - Light	47549	0.22	0	0	0	0.50	0.49	0.50	0.50

Supplementary Table 3. Observed methylated and unmethylated sites for each context in the genome of *P. blakesleeanus*

Context	Methylated sites	Unmethylated sites	Total sites	Enrichment Methylated[a]	Enrichment Unmethylated[a]
CGN	817881	23197813	24015694	203.5	-2,3
CHG	158609	33197105	33355714	-57,6	0,7
CHH	1409960	153889599	15529955	-19,1	0,2

[a]The enrichment was calculated as the deviation between observed and exposed values. Differences were significant (chi-square test p < 0.0001). Two-sided with two degress of freedom

Supplementary Table 4. Genes included in the variable regions between *P. blakesleeanus* NRRL1555 and UBC21

Scaffold	Start	End	ID	Description (KOG)
scaffold_1	1558092	1560029	1819085	Sideroflexin
scaffold_1	1936747	1938541	1949063	Acid sphingomyelinase and PHM5 phosphate metabolism protein
scaffold_1	2862794	2865104	1748008	Permease of the major facilitator superfamily
scaffold_3	2350500	2351462	1970948	
scaffold_3	2723669	2724569	1409181	
scaffold_6	955801	957992	1754815	Predicted oxidoreductase
scaffold_7	2183879	2187075	1928707	
scaffold_8	34283	38934	1928722	
scaffold_8	354025	355438	1928798	
scaffold_8	1729744	1732334	1916879	Uncharacterized conserved protein, contains GRAM domain
scaffold_9	174551	177478	1857138	Membrane-associated guanylate kinase-interacting protein/connector enhancer of KSR-like
scaffold_13	338145	341209	1868994	Translation initiation factor 3, subunit c (eIF-3c)
scaffold_15	416647	417856	1197022	Predicted glycosyltransferase
scaffold_15	416647	417856	1958628	Uncharacterized conserved protein encoded by sequence overlapping the COX4 gene
scaffold_17	332485	333426	1760691	HMG-box transcription factor SOX5
scaffold_17	481032	482155	1797202	
scaffold_17	561602	563225	1797212	
scaffold_18	679541	680534	1933642	DNA helicase PIF1/RRM3
scaffold_24	224643	225958	1798610	

Supplementary Table 5. Genes involved in lipid metabolism in *M. lusitanicus*

Name	*Mucor* ID
SNF-α1	1534495
SNF-α2	1580727
SNF-β	1509430
SNF-γ1	1596935
SNF-γ2	1550946
SNF-γ3	1504902
SNF-γ4	1535000
SNF-γ5	1468593
SNF-γ6	1511039
crtE	1530428
crtE	1504511
carRP	1474672
crtB	1526154
crtI/carB	1474666
bcat1	1522620
bcat2	1476992
bcat3	1489401
hadh	1476825
leuB	1362790
g6pdh1	1455521
g6pdh2	1596585
g6pdh3	1320893
acat1	1483128
acat2	1540408
acat3	1445953
Aldehyde dehydrogenase	1387328
Malate transporter	1462442
Citrate transporter	1595779
Tricarboxylate transporter	1495030
diacylglycerol acyltransferase (DGAT) 1A	1346779
diacylglycerol acyltransferase (DGAT) 1B	1556431
diacylglycerol acyltransferase (DGAT) 2A	1476883
diacylglycerol acyltransferase (DGAT) 2B	1375552
Lip1	1448239
Lip2	1368755
Lip3	1588874
Lip4	1473055
Lip5	1352604
Lip6	1595690
Lip7	1600960
Lip8	1562358
Lip9	1445047
Lip10	1553967
Lip11	1547052
Lip12	1545013
Lip13	1319968
Lip14	1507471
Lip15	1451490
Lip16	1586282
Lip17	1539681
Lip18	1594973
Lip19	1515727
Lip20	1450619
Lip21	1567473
Lip22	1599978
Lip23	1455821
Lip24	1549495
Lip25	1561350
Lip26	1453784
Lip27	1459175
Lip28	1522709
Lip29	1590251
Lip30	1465100

Supplementary Table 6. Genes involved in light sensing regulation in *M. lusitanicus* and *P. blakesleeanus.*

Function	Description	***Phycomyces*** **ID**	***Mucor*** **ID**
BL Photoreception	WC-1	184132	1600988
		77612	1500164
		76314	1584910
	WC-2	77137	1589864
		179643	1485663
		65293	1530145
		85757	1542931
	Cryptochrome	85761	1457883
RL Photorreception	VeA	181799	1557012
	VelB/VelC	106288	1476546
		150993	1459411
		164792	1521819
		185183	1141588
		164792	1377103
		177127	1562578
			1489870
	VosA	185845	1452401
		138522	1324267
		171721	1542853
		162811	1524471
		146331	1545208
		141569	1523399
Carotene biosynthesis	Isopentenyl pyrophosphate dimethylallyl	29590	1551074
	Solanesyl pyrophosphate synthase	133605	1547989
	Farnesyl diphosphate synthase	109964	1465218
		123357	1549377
	Geranylgeranyl pyrophophate synthetase	33722	1504511
		183831	1530428
	Phytoene Synthase/Lycopene cyclase	180114	1474672
		60641	1526154
		59296	
	Phytoene Dehydrogenase	37852	1474666

Supplementary Table 7. Transposons with the highest differential expression

Conditions	TE name	log2FC
WDS/WDM	Polinton1_SM	4,56
	MSAT-5	-4,16
	DNA-1-7_DR	-4,06
	Mariner-18_PI	4,95
WLS/WLM	Mariner-18_PI	4,39
	TcMar-ISRm11	4,54
	P-27_HM	4,71
	DNA3-11_Mad	5,04
	Penelope-5_Hro	5,33
	MULE-MuDR	5,67
	EnSpm-7_Ccri	5,69
	P126	5,91
	Mariner-7_Ror	5,91
	Copia-1_ATe-I	5,96
	Poseidon-2B_PM	6,12
	TLH-1B_Ccri	6,15
	Gypsy18-I_CR	6,39
	Polinton-1_Pba	7,03
	Gypsy2-I_AG	7,58
	Polinton1_SM	7,86
	CR1-8_CQ	8,14
	Helitron-N3_ZM	8,26
	Crypton-F	8,55
	Daphne-73_Hma	8,93
	MuDR-4_Epa	11,05
	MSAT-5	-4,62
	HARB-2_SBi	-3,00
MDS/MDM	Polinton1_SM	4,47
	Tx1-1_CGi	-4,94
	Harbinger-N17_DR	-4,44
MLS/MLM	DNA-27_HM	4,17
	Mariner-18_PI	5,12
	MuDR-4_EPa	6,44
	MSAT-5_DR	4,83

Supplementary Table 8. Strains used in this work

Name	Genotype	Source
M. lusitanicus CBS 277.49	Ura+, Leu+	6
M. lusitanicus NRRL 3631	Ura+, Leu+	NRRL
M. circinelloides WJ11	Ura+, Leu+	7
M. lusitanicus MU636	Ura+, Leu-	8
M. lusitanicus MU1301 (Δ*met1*)	Ura+, Leu-	This Study
M. lusitanicus MU1302 (Δ*met1*)	Ura+, Leu-	This Study
M. lusitanicus MU1303 (Δ*met2*)	Ura+, Leu-	This Study
M. lusitanicus MU1305 (Δ*met2*)	Ura+, Leu-	This Study
M. lusitanicus MU1306 (Δ*met2*)	Ura+, Leu-	This Study
M. lusitanicus MU1307 (Δ*met3*)	Ura+, Leu-	This Study
M. lusitanicus MU1308 (Δ*met3*)	Ura+, Leu-	This Study
M. lusitanicus MU1335 (Δ*mta1*)	Ura+, Leu-	This Study
M. lusitanicus MU1336 (Δ*mta1*)	Ura+, Leu-	This Study
M. lusitanicus MU1337 (Δ*mta1*)	Ura+, Leu-	This Study
M. lusitanicus MU1357 (Δp1)	Ura+, Leu-	This Study
M. lusitanicus MU1358 (Δ*p1*)	Ura+, Leu-	This Study
M. lusitanicus MU1338 (*carRP*::*mta1*)	Ura+, Leu+	This Study
M. lusitanicus MU1339 (*carRP*::*mta1)*	Ura+, Leu+	This Study
M. lusitanicus MU1375 *(carRP*::*mta1* (APPW*)*	Ura+, Leu+	This Study
M. lusitanicus MU1376 *(carRP::mta1(* APPW*)*	Ura+, Leu+	This Study
M. lusitanicus MU1377 *(carRP::mta1* (APPW)	Ura+, Leu+	This Study
M. lusitanicus MU1309 *(ΔΔmet1/met2)*	Ura+, Leu+	This Study
M. lusitanicus MU1310 *(ΔΔmet1/met2)*	Ura+, Leu+	This Study
M. lusitanicus MU1311 *(ΔΔmet1/met2)*	Ura+, Leu+	This Study
P. blakesleeanus NRRL 1555	Ura+, Leu+	
P. blakesleeanus NRRL L51	Ura+, Leu+ (*madA* /*madB*)	9
P. blakesleeanus NRRL UBC21	Ura+, Leu+	

References

1. Amses, K. R. *et al.* Diploid-dominant life cycles characterize the early evolution of Fungi. *Proc. Natl. Acad. Sci. United States Am.* **119**, e2116841119 (2022).

2. Chang, Y. *et al.* Evolution of zygomycete secretomes and the origins of terrestrial fungal ecologies. *iScience* **25**, 104840 (2022).

3. Wang, Y. *et al.* Divergent evolution of early terrestrial fungi reveals the evolution of mucormycosis pathogenicity factors. *Genome Biol Evol* **15**, evad046 (2023).

4. Mondo, S. J. *et al.* Widespread adenine N6-methylation of active genes in fungi. *Nat Genet* **49**, 964–968 (2017).

5. Chaturvedi, A. *et al.* The methylome of the model arbuscular mycorrhizal fungus, *Rhizophagus irregularis*, shares characteristics with early diverging fungi and Dikarya. *Commun Biology* **4**, 901 (2021).

6. Arx, J. A. von & Schipper, M. A. A. The CBS Fungus Collection. *Adv. Appl. Microbiol.* **24**, 215–236 (1978).

7. Tang, X. *et al.* Complete genome sequence of a high lipid-producing strain of *Mucor circinelloides* WJ11 and comparative genome analysis with a low lipid-producing strain CBS 277.49. *PLoS ONE* **10**, e0137543 (2015).

8. Navarro-Mendoza, M. I. *et al.* Early diverging fungus *Mucor circinelloides* lacks centromeric histone CENP-A and displays a mosaic of point and regional centromeres. *Curr. Biol.* **29**, 3791-3802.e6 (2019).

9. Lipson, E. D., Terasaka, D. T. & Silverstein, P. S. Double mutants of *Phycomyces* with abnormal phototropism. *Mol. Gen. Genet. MGG* **179**, 155–162 (1980).

Supplementary Figure 3a – Uncropped scans

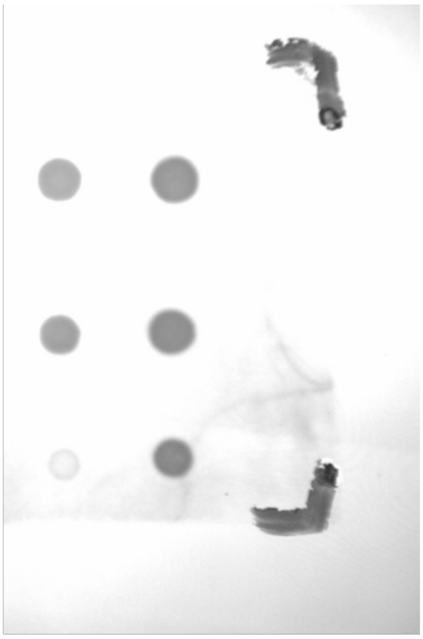

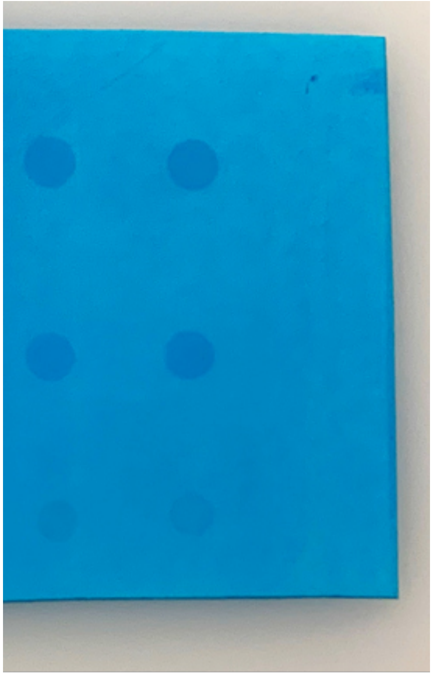

Supplementary Figure 14 – Uncropped gels/blots

metA

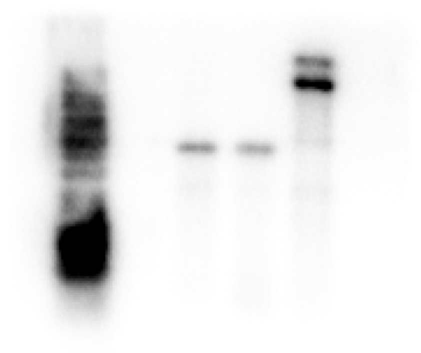

metB

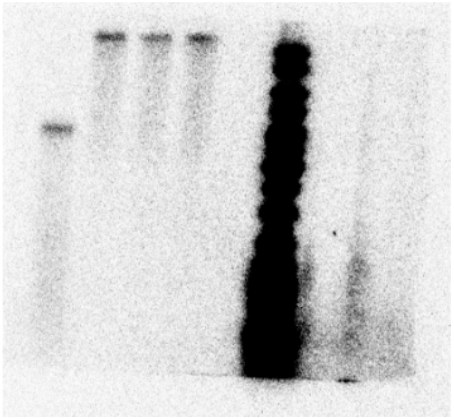

metC

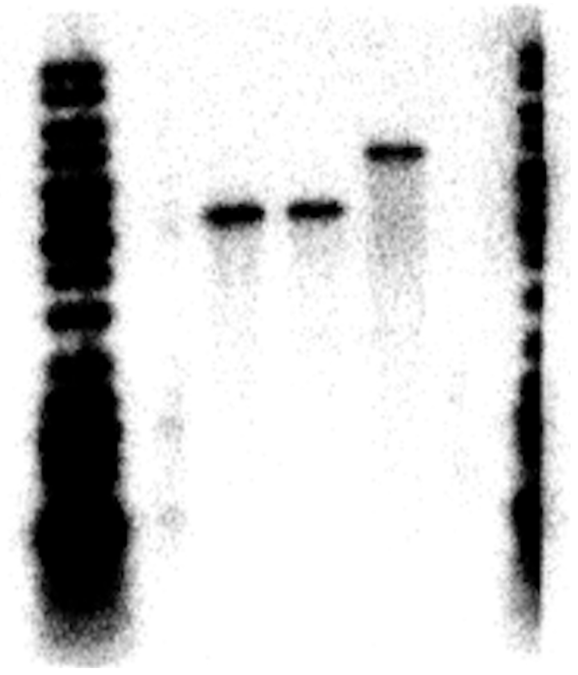

mta1

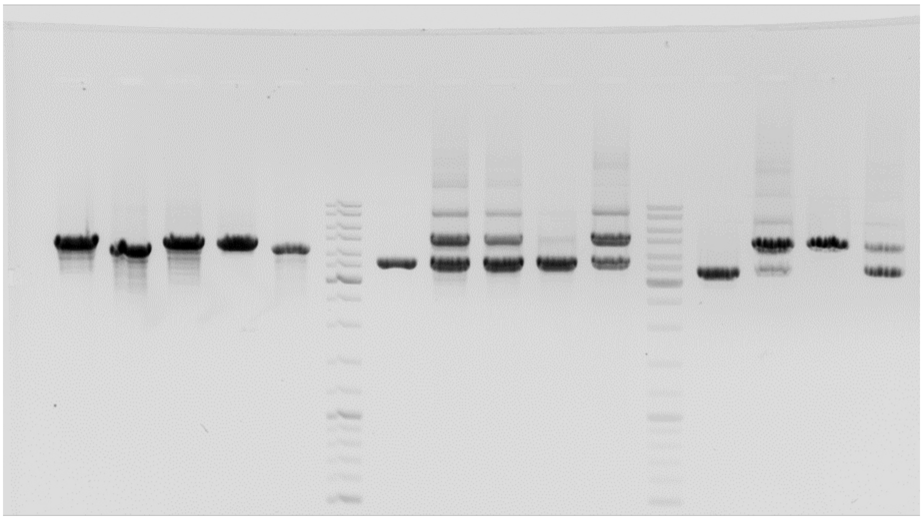

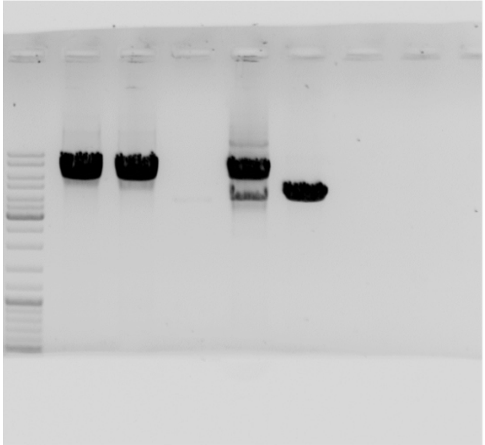

p1

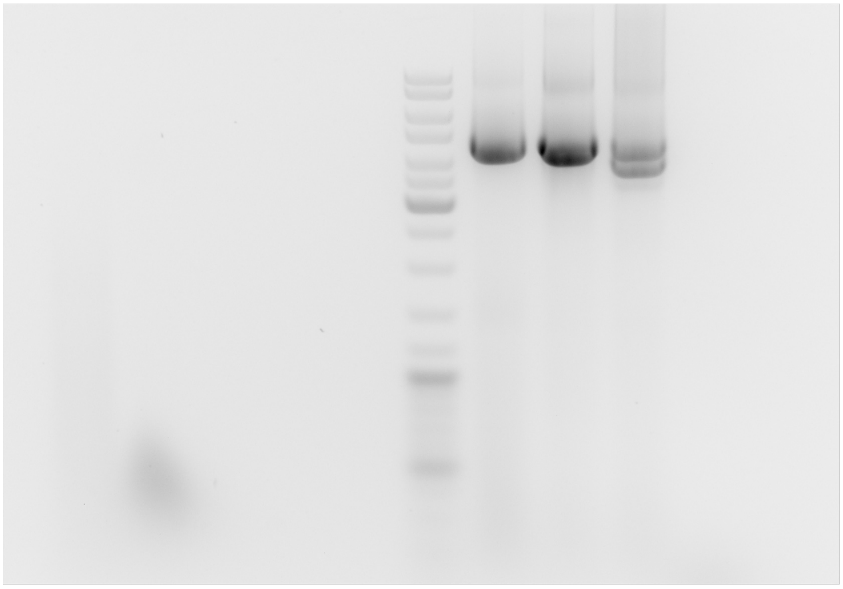

metB – leuA

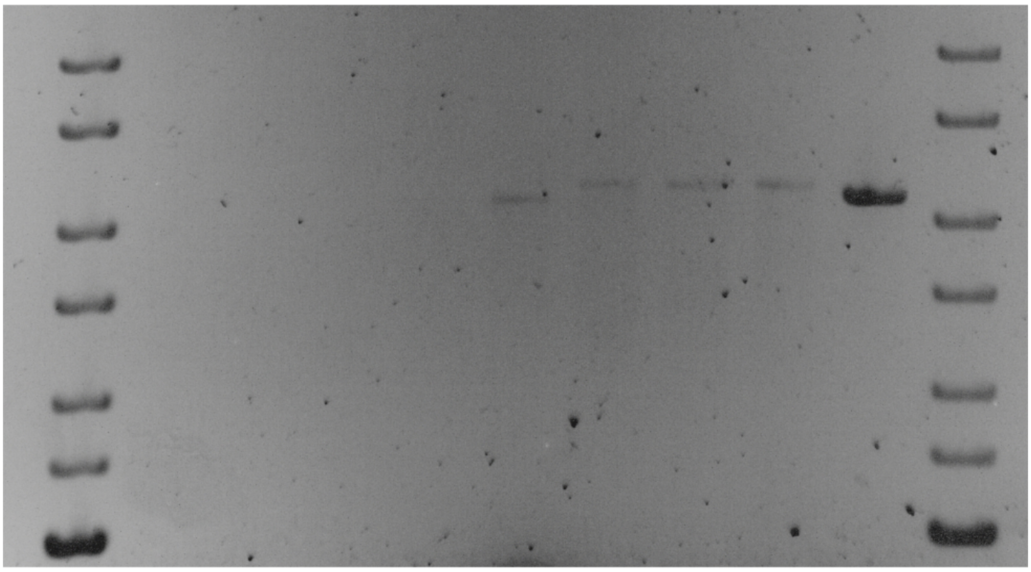

leuA+mta1 complementation

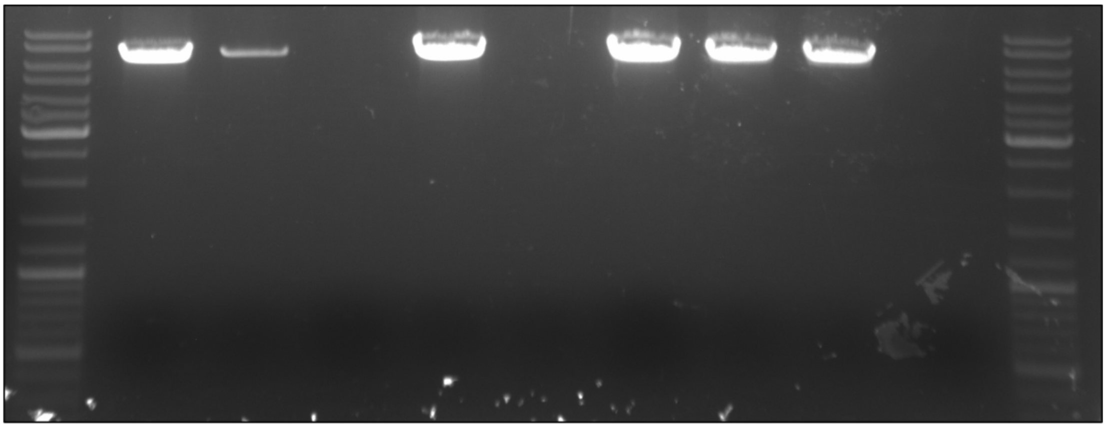

Supplement: Supplementary file 1 — Supplementary Information [file 41467_2024_50365_MOESM1_ESM.pdf]
