# Supplementary material for: Symmetric and asymmetric DNA N6-adenine methylation regulates different biological responses in Mucorales
Source: Nat Commun. 2024 Jul 18;15:6066. doi: 10.1038/s41467-024-50365-2 (PMC11258239; doi:10.1038/s41467-024-50365-2)
Supplement: Supplementary file 3 — Description of Additional Supplementary Files [file 41467_2024_50365_MOESM3_ESM.pdf]

Description of Additional Supplementary Files

Supplementary Data 1. MACs distribution and 6mA content for all conditions tested.

Supplementary Data 2. Differentially expressed genes across all comparisons.

Supplementary Data 3. Number of insertions for each TE as reported by TEfinder for *Phycomyces* NRRL1555 and L51.

Supplementary Data 4. List of genomes analyzed; List of accessions of all fungal homologs; List of copies per genomes analyzed, including short and truncated copies.

Supplementary Data 5. Phylogenetic tree of *metB* homologs with all fungal homologs shown.

Supplementary Data 6. Primers used in this work.

Supplementary Data 7. Statistical test results for genomic 6mA levels showed in Figure 6b.
