# Supplementary figures and images for: Symmetric and asymmetric DNA N6-adenine methylation regulates different biological responses in Mucorales
Source: Nat Commun. 2024 Jul 18;15:6066. doi: 10.1038/s41467-024-50365-2 (PMC11258239; doi:10.1038/s41467-024-50365-2)

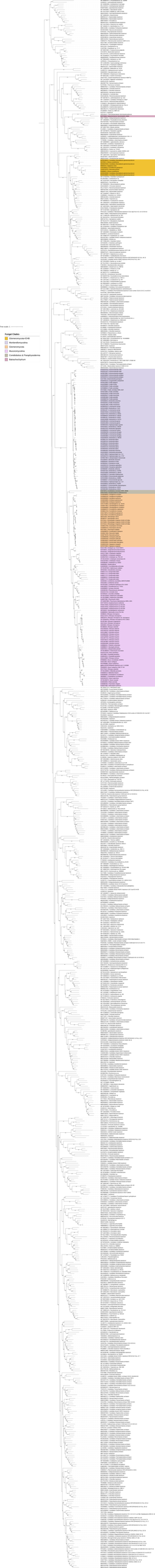

Supplement: Supplementary file 8 — Supplementary Data 5 [file 41467_2024_50365_MOESM8_ESM.pdf]
