# Supplementary material for: Symmetric and asymmetric DNA N6-adenine methylation regulates different biological responses in Mucorales
Source: Nat Commun. 2024 Jul 18;15:6066. doi: 10.1038/s41467-024-50365-2 (PMC11258239; doi:10.1038/s41467-024-50365-2)
Supplement: Supplementary file 11 — Reporting Summary [file 41467_2024_50365_MOESM11_ESM.pdf]

nature portfolio

Corresponding author(s): Luis M. Corrochano
Francisco E. Nicolás
Victoriano Garre

Last updated by author(s): Apr 18, 2024

Reporting Summary

Nature Portfolio wishes to improve the reproducibility of the work that we publish. This form provides structure for consistency and transparency in reporting. For further information on Nature Portfolio policies, see our Editorial Policies and the Editorial Policy Checklist.

Statistics

For all statistical analyses, confirm that the following items are present in the figure legend, table legend, main text, or Methods section.

n/a	Confirmed	
☐	☒	The exact sample size (*n*) for each experimental group/condition, given as a discrete number and unit of measurement
☐	☒	A statement on whether measurements were taken from distinct samples or whether the same sample was measured repeatedly
☐	☒	The statistical test(s) used AND whether they are one- or two-sided<br>*Only common tests should be described solely by name; describe more complex techniques in the Methods section.*
☒	☐	A description of all covariates tested
☐	☒	A description of any assumptions or corrections, such as tests of normality and adjustment for multiple comparisons
☐	☒	A full description of the statistical parameters including central tendency (e.g. means) or other basic estimates (e.g. regression coefficient) AND variation (e.g. standard deviation) or associated estimates of uncertainty (e.g. confidence intervals)
☐	☒	For null hypothesis testing, the test statistic (e.g. *F*, *t*, *r*) with confidence intervals, effect sizes, degrees of freedom and *P* value noted<br>*Give P values as exact values whenever suitable.*
☒	☐	For Bayesian analysis, information on the choice of priors and Markov chain Monte Carlo settings
☒	☐	For hierarchical and complex designs, identification of the appropriate level for tests and full reporting of outcomes
☐	☒	Estimates of effect sizes (e.g. Cohen's *d*, Pearson's *r*), indicating how they were calculated

Our web collection on statistics for biologists contains articles on many of the points above.

Software and code

Policy information about availability of computer code

Data collection	No software was used
Data analysis	JGI Genome Assembly Pipeline which includes: FALCON (v0.5), RACON (v1.4.13), Flye (v2.9-b1768), Juicer (v1.6).<br>Data Analyses with BLASR (v1.5.0), MEME-ChIP(v5.5.5), deepTools (v3.1), MassHunter Workstation Data Acquisition software (vB08.00), HISAT2 (v2.2.0), DESeq2 (v1.30.0), Bismark (v0.24), MethGET, RepeatModeler2 (v2.0.4), RepeatMasker (v4.1.4), ExplorATE (V0.1.0), TEfinder (v1.0.1), IQ-TREE (v2.2.2), MAFFT (v7.508), Orthofinder (v2.5.4), raxmlGUI (v2.0.10), IBM SPSS (v23.0)

For manuscripts utilizing custom algorithms or software that are central to the research but not yet described in published literature, software must be made available to editors and reviewers. We strongly encourage code deposition in a community repository (e.g. GitHub). See the Nature Portfolio guidelines for submitting code & software for further information.

Data

Policy information about availability of data

All manuscripts must include a data availability statement. This statement should provide the following information, where applicable:

- Accession codes, unique identifiers, or web links for publicly available datasets
- A description of any restrictions on data availability
- For clinical datasets or third party data, please ensure that the statement adheres to our policy

The raw sequence data that support the findings of this study have been deposited in the Sequence Read Archive (SRA) under the accession numbers: SRP497170,

SRP497172, SRP497171, SRP497169, SRP497169, SRP496793, SRP496792, SRP496795, SRP496796, SRP496797, SRP496799, SRP496798, SRP496800, SRP496811, SRP496811, SRP496815, SRP496804, SRP496803, SRP496805, SRP496809, SRP496802, SRP496807, SRP496806, SRP496808, SRP496810, SRP496810, SRP496810, SRP496801, SRP496813, SRP496812, SRP496814, SRP496816, SRP496817, SRP496818, SRP496819, SRP496820, SRP496821, SRP496823, SRP496822, SRP496824, SRP496825, SRP496833, SRP496832, SRP496831, SRP496830, SRP496833, SRP496830, SRP496832, and SRP496831. All genomes assembled as part of this study are available through NCBI GenBank (accession number to be provided upon acceptation) and via MycoCosm through the following links: https://mycocosm.jgi.doe.gov/mycocosm/PhyblU21_2 (Phycomyces UBC21), https://mycocosm.jgi.doe.gov/mycocosm/Phybl_L51_1 (Phycomyces L51), and https://mycocosm.jgi.doe.gov/mycocosm/Mucci3 (Mucor CBS277.49).

Research involving human participants, their data, or biological material

Policy information about studies with human participants or human data. See also policy information about sex, gender (identity/presentation), and sexual orientation and race, ethnicity and racism.

Reporting on sex and gender	Not applicable
Reporting on race, ethnicity, or other socially relevant groupings	Not applicable
Population characteristics	Not applicable
Recruitment	Not applicable
Ethics oversight	Not Applicable

Note that full information on the approval of the study protocol must also be provided in the manuscript.

Field-specific reporting

Please select the one below that is the best fit for your research. If you are not sure, read the appropriate sections before making your selection.

☒ Life sciences ☐ Behavioural & social sciences ☐ Ecological, evolutionary & environmental sciences

For a reference copy of the document with all sections, see nature.com/documents/nr-reporting-summary-flat.pdf

Life sciences study design

All studies must disclose on these points even when the disclosure is negative.

Sample size	When conducting both phenotypic and genomic/transcriptomic analyses, we used the sample size following standard procedures from previous publications. Sample sizes were as large as practicable for observations of phenotypes. No statistical methods were used to predetermine sample sizes.
Data exclusions	No data was excluded for the analyses
Replication	RNA-seq samples were triplicated. For phenotypic characterization of growth, sporulation, SDS stress response, virulence, and lipid content all samples were taken by replicates, and all attempts of replication were successful. Results are expressed by mean and SD.
Randomization	The experimental groups were allocated based on the genotype, using appropiate controls for each experiment. Within each experiment group, the samples was randomly selected for statistic analysis.
Blinding	Blinding during data collection was not relevant to most of the experiments conducted. Given that the investigators needed to be aware of both control and treated groups, blinding was not feasible.

Reporting for specific materials, systems and methods

We require information from authors about some types of materials, experimental systems and methods used in many studies. Here, indicate whether each material, system or method listed is relevant to your study. If you are not sure if a list item applies to your research, read the appropriate section before selecting a response.

Materials & experimental systems

n/a	Involved in the study	
☐	☒	Antibodies
☒	☐	Eukaryotic cell lines
☒	☐	Palaeontology and archaeology
☐	☒	Animals and other organisms
☒	☐	Clinical data
☒	☐	Dual use research of concern
☒	☐	Plants

Methods

n/a	Involved in the study	
☒	☐	ChIP-seq
☒	☐	Flow cytometry
☒	☐	MRI-based neuroimaging

Antibodies

Antibodies used	Primary anti-6mA antibody (Synaptic Systems, Cat. No. 202003)
Validation	This antibody has been routinely used in dot blot experiments to detect 6mA with 163 citations according to the manufacturer's website (https://sysy.com/product/202003).

Animals and other research organisms

Policy information about studies involving animals; ARRIVE guidelines recommended for reporting animal research, and Sex and Gender in Research

Laboratory animals	Sixth instar larvae of Galleria mellonella provided by SAGIP (Italy)
Wild animals	The study did not involve wild animals
Reporting on sex	The sex of the larvae was not considered, although it was expected that approximately 50% of the larvae would be of each sex, as no sexual selection was applied.
Field-collected samples	The study did not involve samples collected from the field
Ethics oversight	No ethical approval or guidance was required as Galleria Mellonella is an invertebrate

Note that full information on the approval of the study protocol must also be provided in the manuscript.

Plants

Seed stocks	The study did not involve plants
Novel plant genotypes	The study did not involve plants
Authentication	The study did not involve plants
